# Comprehensive Analysis of Cathepsin Genes in Hemiptera: Functional Characterization of the Venomous Cathepsin B from *Sycanus bifidus*

**DOI:** 10.3390/insects16111078

**Published:** 2025-10-22

**Authors:** Wenkai Liang, Sha Liu, Yuqin Wang, Chaoyan Wu, Wenxiu Wang, Jiaying Zhu

**Affiliations:** 1 Key Laboratory of Forest Disaster Warning and Control of Yunnan Province, Southwest Forestry University, Kunming 650224, China; 1938300828@swfu.edu.cn (W.L.); L3109328917@163.com (S.L.); wangyuqin2022@swfu.edu.cn (Y.W.); wcy1316033@swfu.edu.cn (C.W.); wangwenxiu@swfu.edu.cn (W.W.); 2 Key Laboratory for Forest Resources Conservation and Utilization in the Southwest Mountains of China, Ministry of Education, Southwest Forestry University, Kunming 650224, China

**Keywords:** predatory bug, cathepsin, venom, evolution, melanization

## Abstract

Cathepsins represent a crucial group of protein enzymes involved in insect metabolism. Within the Hemiptera order, comprising a diverse array of predatory, blood-feeding, and herbivorous species, the understanding of cathepsin types and their roles as venom components in predatory bugs remains limited. This investigation systematically identified cathepsin genes present in Hemiptera genomes, highlighting a prevalence of cathepsin B and L, with cathepsin D exhibiting a higher gene count in the Heteroptera suborder. Examining the predatory assassin bug *Sycanus bifidus*, eight cathepsin genes were notably expressed in its venom glands, with the SbCAB2 gene from the cathepsin B subfamily demonstrating the highest expression in the posterior main gland, indicating its significance as a venom component. Subsequent expression and purification of the recombinant SbCAB2 protein revealed heightened hydrolytic activity (0.91 U/mg protein) compared to extracts from the anterior main gland, accessory gland, and gut. Functional assays demonstrated that SbCAB2, at lower doses (0.625–2.5 μg), can impede phenoloxidase activity in *Tenebrio molitor* pupal hemolymph, with a 2.5 μg dose inhibiting 86.5% of this activity, thereby preventing hemolymph melanization. Conversely, a higher dose of 10 μg led to effects akin to human placental cathepsin B, promoting melanization in *T. molitor* pupal hemolymph. These findings lay the foundation for further exploration of the adaptive evolution of cathepsin genes in Hemiptera and offer crucial insights into the functional role of venomous cathepsins in predatory bugs.

## Simple Summary

Cathepsins play crucial roles in insect feeding, digestion, and metabolism. Hemipteran insects exhibit high taxonomic (encompassing species such as bugs, aphids, psyllids, scale insects, and cicadas) and ecological diversity, encompassing phytophagous, hematophagous, and predatory groups; however, knowledge regarding the types and functions of their cathepsins remains limited. In this study, cathepsin genes from 47 hemipteran species were analyzed to systematically investigate the gene types, quantities, and evolutionary relationships of cathepsins within this order. It was found that cathepsins B and L are widely distributed, while cathepsin D is particularly abundant in the Heteroptera suborder. Eight cathepsin genes highly expressed in the venom gland were identified in the predatory reduviid *Sycanus bifidus*, among which the cathepsin B subfamily member *SbCAB2* exhibited the highest expression level in the venom gland. Recombinantly expressed SbCAB2 protein demonstrated high hydrolytic activity and was capable of influencing the phenoloxidase activity in the hemolymph of prey. These findings provide important insights for further investigation into the adaptive evolution of cathepsin genes in Hemiptera and the functional mechanisms of venom cathepsins in predatory reduviids.

## 1. Introduction

Cathepsins, a family of proteases found ubiquitously in the lysosomes of animals, plants, viruses, and human cells, exhibit a propensity for activation under slightly acidic conditions [1]. The concept of cathepsins was first introduced by Willstätter and Bamann [2], derived from the Greek word ‘kathepsein’ associated with digestion. With the successful elucidation of the crystal structure of cathepsin B in the 1990s [3], research on cathepsins has expanded, leading to the identification of a wide array of subfamilies ranging from cathepsin A to X. Based on the amino acids present in the active site, cathepsins can be categorized into cysteine (B, C, L, H, O, S, T, K, V, F), aspartic (D, E), and serine cathepsins (A, G) [4]. According to the cleavage sites of their activity, cathepsins can be further categorized as endopeptidases (D, E, F, G, K, L, S, V) and exopeptidases (A, C, X) [4]. It is noteworthy that cathepsins B and H demonstrate both endo- and exopeptidase activities [4]. Cathepsins are initially inactive zymogens, which are unstable at neutral pH and require specific lower pH conditions within lysosomes for activation through the cleavage of other enzymes to remove propeptides [5]. Their activity can be modulated by protein inhibitors belonging to the cystatin, serpin, and thyropin families, as well as other endogenous protein inhibitors that bind to the active site and prevent substrate hydrolysis [6].

To date, research has predominantly been focused on cysteine cathepsins [7,8,9,10]. Among them, cathepsin B and L have been the primary subjects of research; both exhibited higher activity under weakly acidic conditions (pH 5–6) than in neutral or alkaline environments. In terms of structure, the amino acid sequence of cathepsin B includes six disulfide bridges formed by 12 conserved cysteine residues, as well as an occluding loop of 20 amino acids [11]. This loop is utilized to block peptidase inhibitors, such as cysteine protease, from accessing the enzyme’s active site [12]. The absence of conserved cysteine residues can result in the loss of enzymatic activity in cathepsin B [7]. As for the structure of cathepsin L, it contains a precursor peptide with the inhibitory ERFNIN motif, typically located after the signal peptide [13]. This precursor peptide is cleaved either through intramolecular processing or other enzyme cleavage to produce the mature and active enzyme [14].

Insect cathepsin studies were initiated in 1955 with the identification of cathepsins in the housefly, *Musca domestica*, which were referred to as gastric proteases and presumed to play a role in digestion [15]. In line with other organisms, current research endeavors in insects are predominantly centered around cathepsins B and L [16]. Early investigations suggested their presence as enzymes involved in digestion within the lumen of hemipteran insects [17]. It has been traditionally believed that the primary digestive enzymes in insects are serine endopeptidases. However, evolution-induced selective pressures resulting from environmental changes and other variables have led some insects to incorporate cathepsins within their gut to assist or even surpass serine endopeptidases in facilitating digestion, primarily involving cathepsin B, cathepsin L, and cathepsin D [18]. Beyond their involvement in digestion, they have been recognized to play crucial roles in various physiological processes of insects, including development, metamorphosis, and innate immunity [19,20,21,22,23,24]. However, the evolutionary patterns of cathepsins in insects still require deep elucidation. Furthermore, given their versatility as proteases, uncovering additional physiological functions of cathepsins beyond those already identified remains an area ripe for exploration.

Construction of a cathepsin gene dataset will lay a fundamental foundation for unraveling the evolution as well as biological functions of cathepsin genes. However, only a few studies have been conducted to analyze the cathepsin gene repertoires in a limited number of species in several specific insect orders, providing the considerable evidence that bugs, aphids, and beetles have recruited lysosomal cathepsins as digestive enzymes [18,25,26]. As the largest hemimetabolous insect order, Hemiptera encompasses approximately 103,590 recorded species, with over 10% of all known insect diversity [27,28]. This taxon comprises three groups: Auchenorrhyncha (planthoppers, leafhoppers, spittlebugs, and cicadas), Sternorrhyncha (aphids, scale insects, whiteflies, and psyllids), and Heteroptera (plant bugs, assassin bugs, stink bugs, and lygaeid bugs) [29]. Notably, certain phytophagous species within this order have gained notoriety as agricultural pests (e.g., aphids, scale insects, leafhoppers, and planthoppers) and global medical nuisances (e.g., kissing bugs and bed bugs), while predatory species play crucial roles as natural enemies [30,31]. Given the abundance and diverse habits of hemipterans, which play key roles in agriculture and human health, and considering the biological importance of cathepsins, a thorough investigation and characterization of cathepsins in Hemiptera are essential for elucidating their diversity, evolution, and functions. Despite the extensive hemipteran species diversity, our knowledge of cathepsins within this order remains limited, indicating a gap in understanding that warrants further exploration.

Heteroptera, one of the three suborders of Hemiptera, encompasses the so-called true bugs. This group is prevalent among hemimetabolous insects, with over 42,000 species identified across approximately 90 families to date [32]. Based on their feeding habits, heteropterans can be categorized into herbivorous, predatory, and hematophagous species. They secrete saliva from their salivary glands, which are composed of a main gland divided into the anterior main gland (AMG) and posterior main gland (PMG), as well as an accessory gland (AG) [33]. The saliva is injected into the host plants, prey’s body, or the blood of mammals to facilitate extraoral digestion [34]. This process allows herbivorous bugs to extract nutrients from plant fluids, predatory bugs to obtain nutrients from prey tissues, and hematophagous bugs to feed on mammalian blood [35,36]. The transition from herbivory to predation or hematophagy in Heteroptera distinguishes them from other hemipterans. In predatory or hematophagous bugs, the saliva is also known as venom [37]. The evolution of venom production has been a significant adaptation for heteropterans, enabling them to shift from herbivory to predation and hematophagy, aiding in predation, defense, and feeding [38]. Understanding the venom components and their physiological functions is crucial for comprehending the dietary shift associated with heteropterans, from herbivory to predation and hematophagy. Cathepsins, as well as other diverse types of proteinases such as serine proteinases, lipases, and chitinases, are commonly found as venom components in predatory and hematophagous bugs [36,37,39,40,41,42,43]. However, the biological roles of these proteins as venom components are yet to be fully elucidated.

In this study, cathepsin genes encoded in the genomes of hemipterans were comprehensively identified, followed by an analysis of their phylogenetic relationships. Specifically, detailed exploration of cathepsin genes in the assassin bug *Sycanus bifidus* Fabricius, 1787, which has three synonymous names: *S. croceovittatus* Dohrn, 1859; *S. leucomesus* Walker, 1873; and *S. villicus* Stål, 1863 [44,45], was conducted to elucidate their molecular characteristics and evolutionary trajectories. The expression patterns of these genes in various tissues across different developmental stages were profiled using RNA-seq data and qPCR analysis to gain insights into their potential physiological functions. An exclusive expression of a cathepsin B gene was detected in the salivary venom apparatus of the assassin bug under study. The proteinase encoded by this venom gene exhibited hydrolytic activity implicated in modulating phenoloxidase (PO) activity within the hemolymph of the prey, underscoring its importance in predation. These findings offer significant contributions to the understanding of cathepsin diversity, evolution, and function in hemipterans, particularly the role of cathepsin as a venom constituent in predatory bugs involved in their predation.

## 2. Materials and Methods

### 2.1. Insects

The predatory bug, *S. bifidus*, used in this study was sourced from a laboratory-bred colony that had been cultivated over multiple generations following the methods outlined by Wu et al. [43]. In brief, the first and second instar nymphs were housed in a plastic container and fed a diet of honey water and freshly molted pupae of the yellow mealworm, *Tenebrio molitor*. The third instar nymphs were subsequently relocated to a rearing cage.

Late instar larvae of the yellow mealworm were used as prey for the third to fifth instar nymphs and adults of this bug. The yellow mealworm was raised according to the procedures described by Zhu et al. [46] on wheat bran, with vegetables placed on top of the bran to provide water.

### 2.2. Gene Identification and Sequence Analysis

The amino acid sequences of cathepsin identified in various insects as reported by Silva et al. [26] and those from others deposited in the NCBI Nr database were compiled (Table S1). These sequences were then utilized to search the genome of *S. bifidus* that was sequenced by our laboratory using the Blast tool package within the TBtools-II v. 2.096 [47] with an E-value threshold set at < 1 × 10^−5^, the maximum number of hits retained per query was limited to 500, and the maximum number of alignments per hit was restricted to 250. The potential cathepsin genes identified were subsequently validated by cross-referencing with the Nr database on NCBI using online BLASTp, resulting in the identification of authentic cathepsin genes. Following this method, cathepsin genes were also identified in 47 hemipteran species with available genome data, which were downloaded from InsectBase 2.0 [48,49,50,51,52,53,54,55,56,57,58,59,60,61,62,63,64,65,66,67].

The isoelectric point and molecular weight of these identified cathepsins were predicted using Expasy at https://web.expasy.org/protparam/ (accessed on 20 June 2023). Predictions for signal peptides, structural domains, and N-glycosylation sites were conducted using SignalP-6.0 at https://services.healthtech.dtu.dk/services/SignalP-6.0/ (accessed on 20 June 2023) [68], SMART at https://smart.embl.de/ (accessed on 27 June 2023) [69], and NetNGlyc-1.0 at https://services.healthtech.dtu.dk/services/NetNGlyc-1.0/ (accessed on 27 June 2023) [70], respectively. Multiple sequence alignments were performed using ClustalX v. 1.83 software [71] with the global complete alignment mode and default parameters. The results were exported in MSF format, and the alignment results were colorized using GeneDoc v. 2.6.02 software. To remove the randomized sequence regions from the multiple sequence alignment, trimming was performed using AliCut v. 2.31 at https://github.com/PatrickKueck/AliCUT (accessed on 28 June 2023). Subsequently, a phylogenetic tree was constructed using the maximum likelihood method with the IQtree2 plugin in TBtools-II v. 2.096 [47,72], with the best-fit evolutionary model automatically selected and the bootstrap number set to 1000. The phylogenetic tree was visualized using FigTree v. 1.4.4 [73].

### 2.3. Gene Expression Profiling

Various tissues from different developmental stages of *S. bifidus*, as specified in Section 3, were dissected in phosphate buffer under a stereomicroscope following the protocol outlined by Su et al. [74]. The dissected tissues were then collected and subjected to total RNA extraction using Trizol reagent (Invitrogen, San Diego, CA, USA) according to the manufacturer’s instructions. The integrity, concentration, and purity of the total RNA were evaluated through 1% agarose gel electrophoresis, NanoDrop ND-1000 spectrophotometer (NanoDrop Technologies, Rockland, DE, USA), and Agilent Bioanalyzer 2100 system (Agilent Technologies, CA, USA).

For Illumina RNA-seq sequencing, the methodology was outlined in detail by Wu et al. [43]. The clean data previously obtained [43] for the tissues were aligned to the reference genome of *S. bifidus* using HISAT v. 2.2.2.1 [75]. The read counts resulting from the alignment were utilized to compute the TPM (transcripts per million) values using DESeq2 v. 1.28.1 [76]. Subsequently, the TPM values corresponding to the identified cathepsin genes were extracted. Using the HeatMap package in TBtools-II v. 2.096 software, Log_2_ (TPM + 1) transformation was applied to handle zero values. The resulting log-transformed values were then normalized to a 0–1 range using min–max scaling to visualize expression trends of each gene across different samples [47].

For the quantitative real-time PCR (qPCR) analysis, cDNA templates were synthesized using the PrimeScript RT Reagent Kit with gDNA Eraser (TaKaRa, Dalian, China) following the provided instructions, utilizing 1 µg of total RNA extracted from each tissue. Gene-specific primers, as detailed in Table S5, were designed using Beacon Designer 8.21 software (PREMIER Biosoft, San Francisco, CA, USA) based on the sequences of the previously identified cathepsin genes. The 40S ribosomal protein S3-A (*SbRPS3A*) gene served as the reference gene. qPCR experiments were conducted with the Bestar^®^ SybrGreen qPCR Mastermix (DBI Bioscience, Shanghai, China) to quantify the expression levels of each gene in various tissues, using a qTOWER 2.2 Real-Time qPCR Thermal Cycler (Analytik Jena AG, Jena, Germany). The PCR conditions included an initial denaturation step at 95 °C for 2 min, followed by 40 cycles of 95 °C for 10 s, 58 °C for 31 s, and 72 °C for 30 s. Each sample was analyzed with three biological replicates and three technical replicates. The qPCR data analysis was performed using the Q-gene method [77].

### 2.4. Recombinant Production of Protein

Based on the previously identified SbCAB2 gene sequence, the cDNA of its open reading frame was synthesized accurately through a PCR-based protocol [78]. This specific sequence excluded the region encoding the signal peptide, but included *BamH I* and *Xho I* termini. After validating the PCR product via Sanger sequencing, it was cleaved with *BamH I* and *Xho I* before being cloned into the pFast-bac1 vector from the Bac-to-Bac^®^ Vector Kit (Thermo Fisher Scientific, Waltham, MA, USA). The recombinant plasmid was then transformed into DH10Bac competent cells (Invitrogen, San Diego, CA, USA). Positive colonies were selected, and recombinant bacmids were extracted. The correctness of the PCR products was confirmed again by Sanger sequencing. This generated the bacmid used for Sf9 cell transfection using Cellfectin II Reagent (Thermo Fisher Scientific, Waltham, MA, USA). The cells were grown in Sf-900 II SFM media (Thermo Fisher Scientific, Waltham, MA, USA). For P1 virus production, 2 mL of Sf9 cells at an approximate concentration of 2.0 × 10^6^ cells/mL were used. The expression of the resulting recombinant protein was verified via Western blotting with a His-tag antibody. For P2 virus amplification, 30 mL of Sf9 cells at a density of 2.0 × 10^6^ cells/mL were inoculated into a flask and infected with P1 virus, followed by incubation at 27 °C with shaking at 120 rpm for 3–5 days. The culture was subsequently centrifuged, and the supernatant was collected.

The protein product was purified using Ni-NTA Sepharose 6FF (Solarbio, Beijing, China), following the manufacturer’s instructions, and analyzed using 10% SDS-PAGE gel electrophoresis for purity confirmation. The purified protein underwent dialysis in a PBS buffer (pH 7.4), then storage in DNase/RNase-free 1.5 mL centrifuge tubes. The protein concentration was determined by the Bradford method [79] before being stored at −80 °C until analysis.

### 2.5. Assay of Cathepsin Activity

The PMG, AMG, AG and gut were dissected from *S. bifidus* adults as previously mentioned, and placed into a 1.5 mL centrifuge tube containing 100 μL of PBS (pH 7.4). After being thoroughly ground using a grinding rod, the mixture was centrifuged at 4 °C and 12,000 g for 10 min. The supernatant was transferred to another tube to conduct an enzyme activity assay, and the protein concentration was measured using the Bradford method [79]. These proteins served as positive controls, and bovine serum albumin (BSA) (Shanghai Shenggong Biological Co., Ltd., Shanghai, China) was used as a negative control to determine the activity of cathepsin B in the recombinant SbCAB2. The cathepsin B activity assay followed the method of Saito et al. [80], with minor modifications, using Z-Arg-Arg-MCA (Shenzhen Jianzhu Technology Co., Ltd., Shenzhen, China) as the substrate. The standard curve was established using human placental cathepsin B (Shenzhen Jianzhu Technology Co., Ltd., Shenzhen, China).

In a 1.5 mL centrifuge tube, 20 μL of recombinant SbCAB2, enzyme solution of the venom from PMG, AMG and AG as well as gut, and BSA, were added along with 200 μL of freshly prepared buffer (containing 58.44 mg of sodium chloride, 2.9325 mg of EDTA, 10.4 mg of disodium hydrogen phosphate, 148.4 mg of monosodium phosphate, 50 μL of 10 mM substrate, and 3.5 μL of 2-mercaptoethanol in 10 mL of ddH_2_O). The reaction solution was incubated at 27 °C for 10 min, followed by an immediate quenching via the addition of 200 μL of 20% ethanol. Fluorescent absorbance was then measured with a fluorescence spectrophotometer (RF-5301PC, Shimadzu, Japan) set in ‘Quantitative’ mode at excitation and emission wavelengths of 380 nm and 460 nm, respectively. The enzymatic activity was then calculated in units (U) as per the method described by Dai et al. [81]. Here, a single unit of protease activity is defined as the quantity of enzymatic protein needed to hydrolyze 1 μmol of substrate within a timeframe of 10 min under the above specified experimental conditions.

### 2.6. Assay of Phenoloxidase Activity

Hemolymph from newly pupated *T. molitor* pupae was collected by puncturing the wing buds into a sterile 1.5 mL centrifuge tube, followed by centrifugation at 4 °C, 3000 rpm for 10 min. The resulting plasma supernatant was transferred to a new tube. Protein concentration in the plasma was assessed using the Bradford method [79]. Subsequently, 2 µL of plasma (40 µg/µL) was combined with 87.5 µL of recombinant SbCAB2 at different doses (10 µg, 5 µg, 2.5 µg, 1.25 µg, and 0.125 µg) in a centrifuge tube, followed by a 10 min incubation at 25 °C. A 0.02 M L-DOPA solution, prepared by dissolving 3.94 mg in 1 mL of 0.9% NaCl solution, was then added to reach a final volume of 300 µL. After thorough mixing, 200 µL of the mixture was transferred to a 96-well plate for absorbance measurement at 490 nm using a microplate reader (TECAN, Männedorf, Switzerland) at 5 min intervals over 60 min. A 0.02 M propylthiouracil (PTU) solution and 87.5 µL BSA (10 µg) served as positive and negative controls, respectively. Each sample was assayed in three technical replicates. Enzymatic activity was quantified in units (U), where one unit of PO activity was defined as 0.001 ΔA_490_/min, following the method described by Yan et al. [82].

### 2.7. Statistical Analysis

The data shown in the figures were subjected to Tukey’s tests utilizing GraphPad Prism 8.0 software (GraphPad Software, San Diego, CA, USA). Statistical significance was defined as a *p* value less than 0.05.

## 3. Results

### 3.1. Cathepsin Gene Repertoires in Hemipteran Species

**Figure 1:**
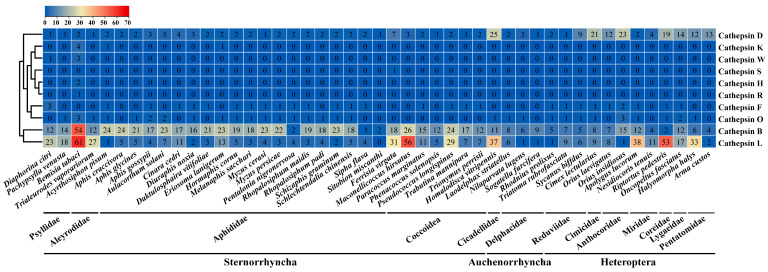
Heatmap of cathepsin gene quantity distribution and classification in hemipteran species. The color intensity in the heatmap represents the number of cathepsin genes across different species, and the scale bar illustrates the corresponding relationship between color and numerical values.

A comprehensive analysis of cathepsin gene repertoires was conducted in the incorporating species from three Hemiptera suborders, each with a high-quality completed genome. A total of 47 species from 13 families were examined. This led to the identification of 1744 unique cathepsin protein sequences (Figure 1 and Tables S2 and S3). Specifically, from the suborder Sternorrhyncha, we identified 1085 cathepsins in two psyllids, two whiteflies, 20 aphids, and seven mealybugs. From the suborder Auchenorrhyncha, 131 cathepsins were identified in one leafhopper and three planthoppers. Lastly, from the suborder Heteroptera, we discovered 528 cathepsins in a group of 12 true bugs.

Of the cathepsins identified, ten subfamilies were found including cathepsins B, D, L, F, O, K, W, H, R, and S. All analyzed hemipterans possessed cathepsins from families B and D. Cathepsins from the L subfamily were also prevalent across all hemipterans, except for the grain aphid, *Sitobion miscanthi*. The majority of species held cathepsins from subfamilies F and O, though the gene numbers were relatively low, less than four. In contrast, cathepsins from subfamilies K, W, H, R, and S were identified in only a handful of species and were reflected in no more than five gene numbers. Notable variability was observed in the gene number identified for each species, ranging from three in *S. miscanthi* to 130 in the invasive whitefly, *Bemisia tabaci*. However, the majority of species possessed cathepsin gene numbers within the range of 20 to 40. Within each species, the gene repertoires were majorly composed of cathepsins from subfamilies B, D, and L (Figure 1).

Fewer genes from cathepsin B were identified in species from the Delphacidae, Reduviidae, Cimicidae, and Pentatomidae families, but genes from this family are abundantly found in most other hemipteran species, with the number often exceeding ten. Most aphid species have a low number of identified cathepsin L genes, usually fewer than ten, whereas in most other hemipteran species, the number of genes from this family typically exceeds ten. Interestingly, most true bugs displayed a larger repertoire of cathepsins from the D subfamily, ranging from 4 to 23. However, the count of cathepsins within this category was generally less than five in most other species. A notable exception was the glassy-winged sharpshooter, *Homalodisca vitripennis*, which distinctively possessed 25 genes from this family (Figure 1).

### 3.2. Phylogenetic Analysis of Cathepsin Genes

**Figure 2:**
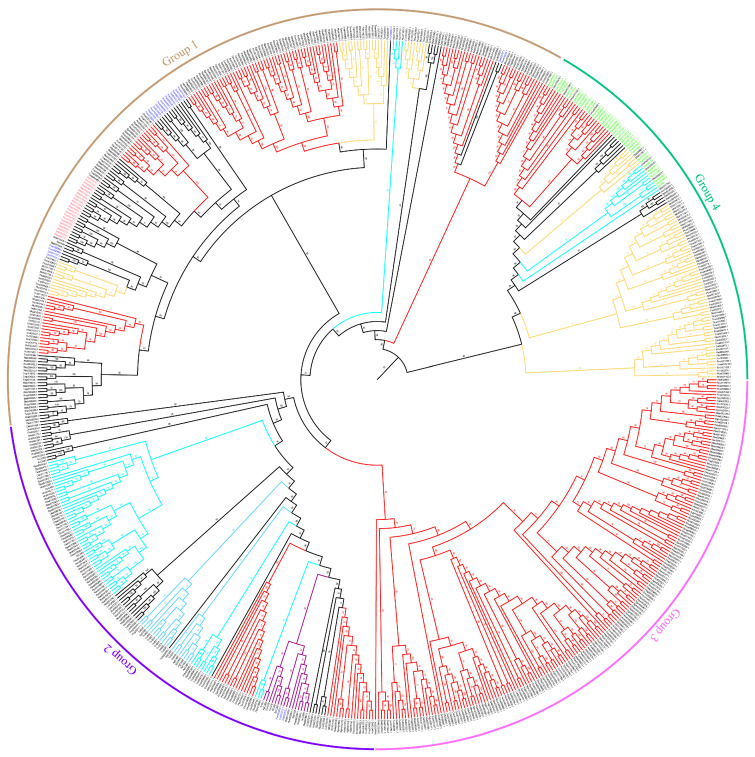
Phylogenetic tree of cathepsin B proteins. Gene names are color-coded as follows: purple for Psyllid cathepsin B; green for cathepsin B containing the Somatomedin_B structural domain; and pink for *Bemisia tabaci* cathepsin B. Branches in different colors indicate: red for aphid cathepsin B; yellow for Coccidae cathepsin B; cyan for Heteroptera cathepsin B; dark purple for lysosomal cathepsin B; and light blue for non-lysosomal cathepsin B. For species names in the figure, refer to the list of abbreviations.

In the Hemiptera order, cathepsins were subjected to phylogenetic analysis alongside cathepsins from other insects, including *Anoplophora glabripennis* and *T. molitor* from Coleoptera, and *Bombyx mori* and *Spodoptera litura* from Lepidoptera, as well as *Aedes aegypti* and *Drosophila melanogaster* from Diptera. The cathepsins B from Heteroptera insects (cyan branch) formed a well-supported separate branch distinct from Aphididae (red branch) and Coccoidea (yellow branch), which included both lysosomal (dark purple branch) and non-lysosomal cathepsins B (light blue branch). Notably, cathepsins B showed gene expansion in the Aphididae compared to other species. Conversely, cathepsins B from Coccoidea were dispersed in various branches. Psyllidae cathepsins B (purple branch) were fewer and exhibited clustering with lysosomal cathepsins B, indicating their lysosomal nature. Cathepsins B from Hemiptera suborder, Aphididae, and Coccoidea, possessing the Somatomedin_B domain, clustered together in a distinct branch (Figure 2).

**Figure 3:**
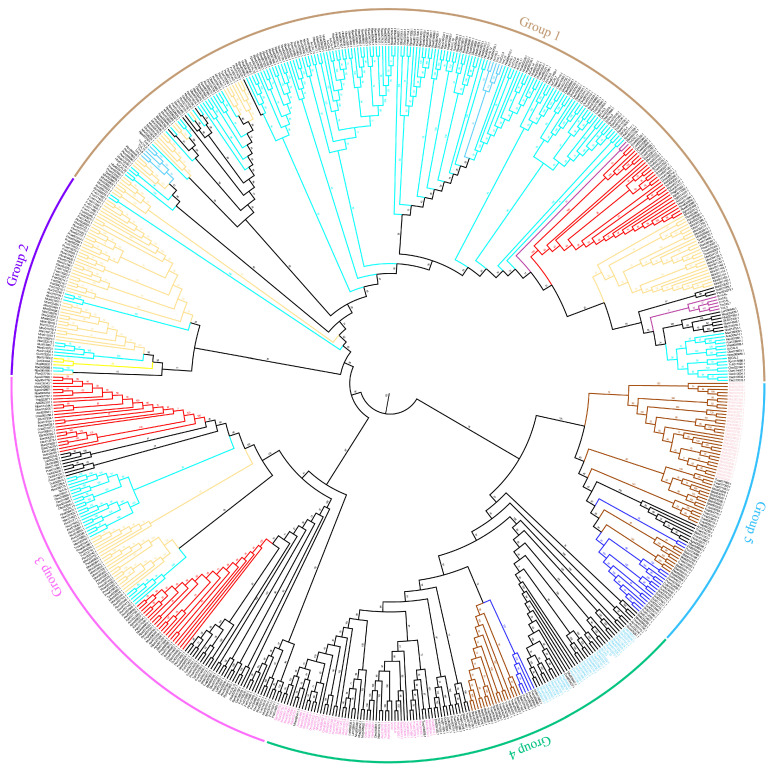
Phylogenetic tree of cathepsin L proteins. Gene names are color-coded as follows: purple for Psyllidae cathepsin L; pink for *Bemisia tabaci* cathepsin L; light purple for cathepsin F; and light blue for cathepsin O. Branches in different colors indicate: red branch for Aphididae cathepsin L; yellow branch for Coccidae cathepsin L; cyan branch for Heteroptera cathepsin L; dark purple branch for lysosomal cathepsin L; light blue branch for non-lysosomal cathepsin L; navy blue branch for Psyllidae cathepsin L; and brown branch for Aleyrodidae cathepsin L. For species names in the figure, refer to the list of abbreviations.

Cathepsins L from Heteroptera grouped (cyan branch) with non-lysosomal cathepsins L (light blue branch), forming distinct clusters. Cathepsins L from Psyllidae (purplegene name and navy blue branch) and Aleyrodidae (brown branch) clustered closely, suggesting a close relationship. In contrast, cathepsins L from Aphididae (red branch) and Coccoidea (yellow branch) did not form distinct clusters and were dispersed with low support among other branches (Figure 3).

**Figure 4:**
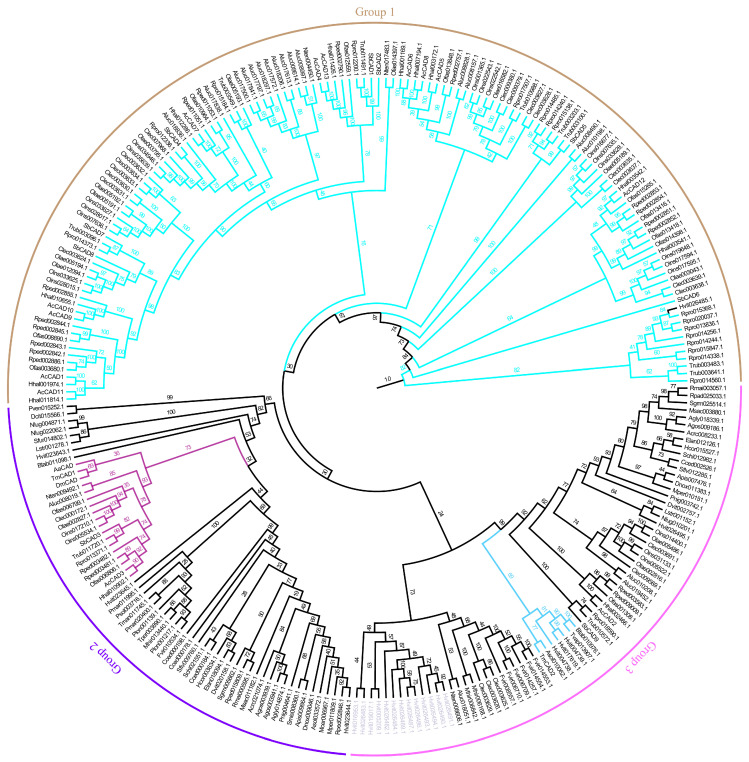
Phylogenetic tree of cathepsin D proteins. Light purple gene name represents cathepsin D of *Homalodisca vitripennis*. Branches in different colors indicate: cyan for Heteroptera cathepsin D; dark purple for lysosomal cathepsin D; and light blue for non-lysosomal cathepsin D. For species names in the figure, refer to the list of abbreviations.

Cathepsins D from Heteroptera (cyan branch) exhibited a greater number compared to the suborders Auchenorrhyncha and Sternorrhyncha, forming a distinct cluster indicative of a gene expansion event. Cathepsins D from *Homalodisca vitripennis* (light purple gene name) formed a separate branch distinct from cathepsins D of other species (Figure 4).

### 3.3. Molecular Characteristics of S. bifidus Cathepsins

A total of 22 cathepsins, represented by five subfamilies, were decoded from the *S. bifidus* genome. This set includes cathepsins B (SbCAB1–SbCAB5), D (SbCAD1–SbCAD9), L (SbCAL1–SbCAL6), F (SbCAF), and O (SbCAO) (Table 1 and Table S4). The predicted molecular weights of these cathepsins span from 22.23 kDa (SbCAD6) to 89.94 kDa (SbCAF), and their isoelectric points fall within the range of 4.95 (SbCAD4) to 9.54 (SbCAD6). Within them, all except SbCAB3, SbCAB4, SbCAD5, SbCAD8, SbCAL2, and SbCAL5, contain a signal peptide (Table 1). Notably, only SbCAD1, SbCAL3, and SbCAL6 do not possess N-glycosylation sites, whereas the other cathepsins have vary counts of such sites ranging from one to six (Table 1).

Variations were observed in the catalytic residues of cathepsins B, D, L, F, and O in *S. bifidus* during the multiple sequence alignment (Figures S1–S3). Notably, in SbCAL6, the active site’s catalytic residues Cys and His were substituted by Ser and Arg, respectively. In SbCAB4, the catalytic residue His was replaced by Leu at the active site. However, the catalytic residues in other cysteine proteases (SbCAL1–5, SbCAB1, SbCAB2, SbCAB3, SbCAB5, SbCAF, and SbCAO) remained conserved as Cys, His, and Asn residues at their active sites. Concerning the aspartic proteases, SbCAD1–5, SbCAD7, and SbCAD9 all exhibited two conserved Asp residues. In SbCAD8, the second Asp residue at the active site was substituted with a Ser residue. Notably, SbCAD6 did not display the typical conserved motifs and catalytic residues characteristic of the aspartic protease family (Figure S2).

The alignment results of multiple amino acid sequences of cysteine proteases, including cathepsins L, O and F, revealed that the motif sequences of ERFNIN and GNFD are conserved among cathepsin L members (Figure S1). Neither of these motif sequences is found in cathepsin O and F. Cathepsin O contains only a portion of the ERFNIN sequence. Meanwhile, the DTGS and DTGT motif sequences, which are characteristic of aspartic proteases, are conserved in cathepsin D members (Figure S2). In cathepsins B, the occluding loop has been identified (Figure S3). SbCAB2, SbCAB3, and SbCAB5 exhibit an intact closed loop, which incorporates two crucial histidine residues. However, SbCAB1 has only a single histidine residue, and SbCAB4 does not contain any histidine residues.

**Figure 5:**
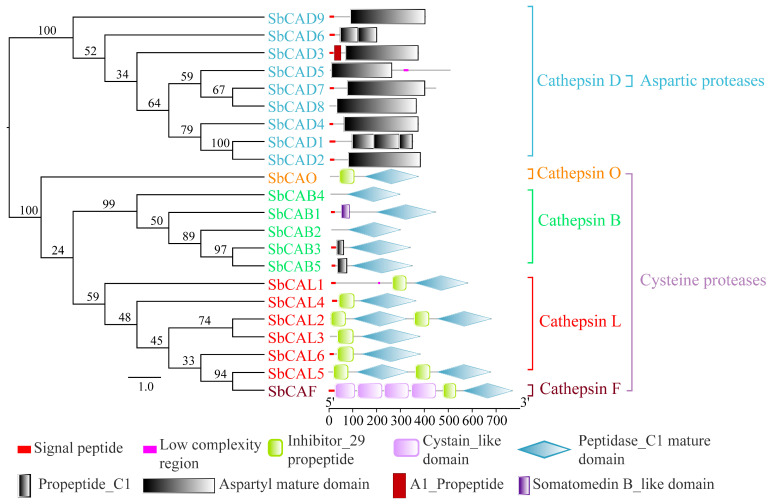
Phylogenetic and structural domain analysis of cathepsin of the *Sycanus bifidus*. Different shapes of color blocks indicate different structural domains.

Of the identified cathepsins, the propeptide_C1 mature domain was absent in the D subfamily, but the peptidase_C1 mature domain present across all the remaining cathepsins from the B, L, O, and F subfamilies (Figure 5). The Aspartyl mature domain was present in the D subfamily, but not in other subfamilies. SbCAD6 possessed two of these domains, SbCAD1 had three, and all other members had one such domain. Moreover, the inhibitor_I29 domain was observed in the cathepsins of the L, O and F subfamilies, but it was absent in those of the D and B subfamilies. Interestingly, SbCAB1 was found to have a Somatomedin_B-like domain, and four serial cystatin-like domains were identified in SbCAF.

Analysis of the cathepsin genes located on the chromosome revealed that SbCAD1 and 2, SbCAD5–8, and SbCAL2–6 genes were closely clustered (Figure S4). However, the other cathepsin genes were not as tightly consolidated. Manual inspection confirmed that no genes were located between these closely grouped genes. The phylogenetic tree also demonstrated that these clustered genes from each subfamily fell into relatively smaller groups (Figure 3 and Figure 4). These suggest that the origin of these genes is a result of gene duplication.

### 3.4. Profiling the Expression of Cathepsin Genes in S. bifidus

**Figure 6:**
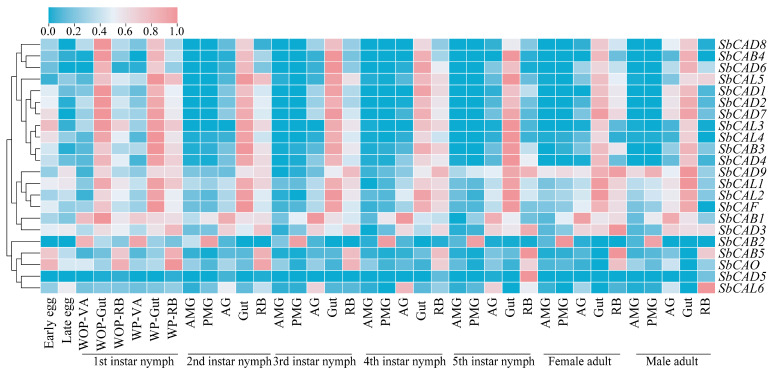
Gene expression profiles of cathepsins in different tissues and developmental stages of *Sycanus bifidus*. The transcript levels of cathepsin genes (expressed as Log_2_-transformed TPM values) were visualized in the heatmap. In this representation, deep red indicates higher transcript levels, while blue denotes lower transcript levels. WOP, 1st instar nymphs without predation ability; WP, 1st instar nymphs with predation ability; VA, venom apparatus; AMG, anterior main gland; PMG, posterior main gland; AG, accessory gland; RB, residual body, bugs deprived of venom apparatus and gut.

To understand the potential functions of the *S. bifidus* cathepsin genes, their transcriptions in various tissues during different developmental stages were profiled using RNA-seq data (Figure 6). The results indicated that most cathepsin genes exhibit high levels of expression in eggs. Across life stages, spanning from the first instar nymph to adult, cathepsin genes, excluding *SbCAB2*, *SbCAB5*, *SbCAD5*, *SbCAL6,* and *SbCAO*, are highly expressed in the gut. Most of these cathepsin genes, which demonstrate abundant transcription in the gut, also exhibit considerable expression in residual bodies, though lower than that in the gut and with comparable levels maintained from the first instar nymph stage through to adult. Moreover, genes displaying high levels of expression in the AMG, PMG, and AG maintain these elevated levels across the developmental stages from egg to adult. Apart from a small number of cathepsin genes, most show relatively high levels of expression in the AG, with *SbCAB1* and *SbCAD3* recording the highest transcription levels. With regard to the AMG, only *SbCAD9* displayed high expression. In the PMG, *SbCAB1*, *SbCAB2*, and *SbCAD9* demonstrated high levels of expression, with *SbCAB2* being specifically expressed in this tissue.

### 3.5. Validating the Expression of Cathepsin Genes in S. bifidus

**Figure 7:**
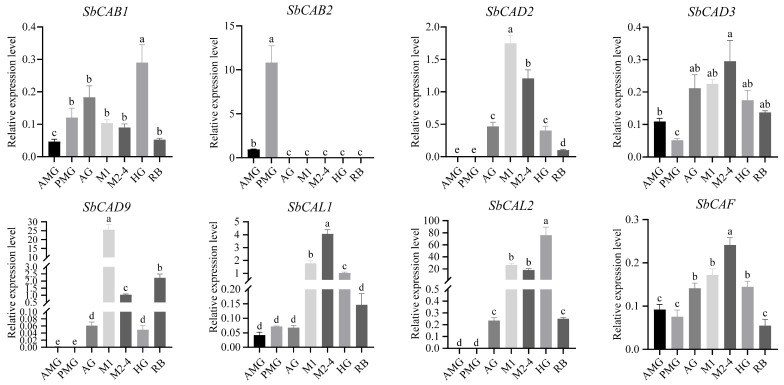
Expression patterns of venom cathepsin genes across adult tissues in *Sycanus bifidus* validated by qPCR. AMG, anterior main gland; PMG, posterior main gland; AG, accessory gland; M1, midgut 1; M2–4, midgut 2–4; HG, hindgut; RB, residual body, bugs deprived of venom apparatus and gut. The bars indicate the mean ± standard deviation (SD). A one-way ANOVA followed by Tukey’s test was conducted, with different lowercase letters denoting significant differences between tissues (*p* < 0.05).

Based on the gene expression profiling results obtained from RNA-seq data, several potential venomous cathepsin genes displaying high expression in three distinct glands, AMG, PMG, and AG, have been selected. Their expression levels in various tissues were further validated through qPCR (Figure 7). The chosen genes are as follows: *SbCAB2*, observed exclusively in PMG; *SbCAB1*, predominantly expressed in AG, along with *SbCAD2*, *SbCAD3*, *SbCAD9*, *SbCAL1*, *SbCAL2*, and *SbCAF*, manifesting relatively high expression in AG. As per qPCR findings, the expression patterns of the selected genes agree with those derived from the RNA-seq data profiling. These findings suggest that the cathepsin genes exhibiting high expressions in venom glands could be encoding the protein components specific to the gland in which they are expressed. Notably, SbCAB2 gene exhibits a particularly high expression level in PMG, thus suggesting that it is a venom cathepsin gene responsible for encoding a substantial protein component within PMG.

Cathepsin B exhibits the highest number of gene expansions among 47 hemipteran species. In phytophagous species, cathepsin B serves as a key effector in feeding and digestion processes [25]. Meanwhile, cathepsin B genes are present in relatively high numbers in predatory stink bugs, suggesting their potential role in venom secretion and prey capture. Therefore, through transcriptome sequencing and qPCR validation, *SbCAB2*, which shows the highest expression abundance in the venom gland, was selected as a candidate gene for further analysis of its biological functions.

### 3.6. Assay of the Enzymatic Activity of the Recombinant SbCAB2

**Figure 8:**
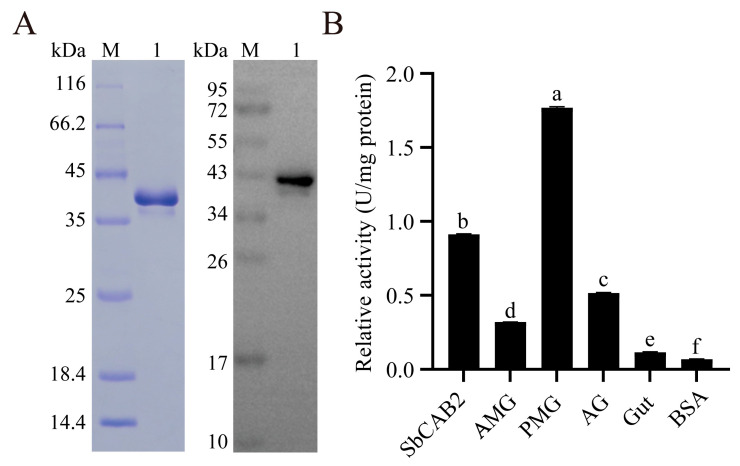
Expression purification and enzyme activity assay of SbCAB2. (**A**) SDS-PAGE and Western blot analysis of purified SbCAB2. Lane M, protein marker; Lane 1, purification of extracellularly secreted SbCAB2. (**B**) SbCAB2 enzyme activity assay. SbCAB2, purified SbCAB2; AMG, anterior main gland extracts; PMG, posterior main gland extracts; AG, accessory gland extracts; Gut, gut extracts; BSA, bovine serum albumin. Data represent the mean ± SD. Significant differences are denoted by different letters, as determined by one-way ANOVA followed by Tukey’s test (*p* < 0.05).

The SbCAB2 gene was efficiently expressed using the baculovirus-insect cell expression system, and the resulting protein was purified via a Ni-NTA Beads 6FF affinity chromatography column. SDS-PAGE and Western blot analysis indicated that the molecular weight of the recombinant SbCAB2 protein was approximately 37 kDa (Figure 8A). With Z-Arg-Arg-MCA serving as a substrate, the recombinant SbCAB2 displayed high enzymatic activity, detected at 0.91 U/mg protein (Figure 8B). This activity exceeds the cathepsin activity found in gut extraction, AMG venom, and AG venom but is lower than the cathepsin activity observed in PMG venom.

### 3.7. Effects of Recombinant SbCAB2 on Hemolymph Melanization

**Figure 9:**
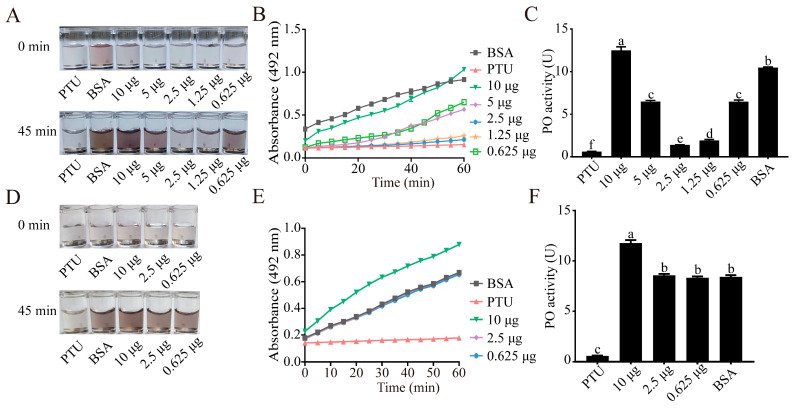
Effect of SbCAB2 on hemolymph melanization in *Tenebrio molitor*. (**A**) Colorimetric reaction of hemolymph-SbCAB2 solution at 0 min and 45 min. (**B**) Absorbance changes in hemolymph-SbCAB2 reaction solution recorded over 0–60 min. (**C**) Inhibitory effect of varying doses of SbCAB2 on PO activity in *T. molitor* hemolymph. (**D**) Colorimetric reaction of hemolymph-human placental cathepsin B solution at 0 min and 45 min. (**E**) Absorbance changes in hemolymph-human placental cathepsin B reaction solution recorded over 0–60 min. (**F**) Inhibitory effect of varying doses of human placental cathepsin B on PO activity in *T. molitor* hemolymph. Phenylthiourea (PTU) was used as a positive control and BSA solution as a negative control. Data represent the mean ± SD. Significant differences are denoted by different letters, as determined by one-way ANOVA followed by Tukey’s test (*p* < 0.05).

To investigate the impact of SbCAB2 on the immune response of prey, the influence of different concentrations of SbCAB2 on melanization in *T. molitor* pupal hemolymph was assessed (Figure 9A–C). Results revealed that at 0.625 μg, SbCAB2 led to a 39.0% inhibition of PO activity in the pupal hemolymph of *T. molitor*, leading to the inhibition of hemolymph melanization. Increasing concentrations of 1.25 μg and 2.5 μg of SbCAB2 resulted in higher inhibition rates of 82.0% and 86.5% on PO activity, respectively. However, the inhibitory effect diminished at 5 μg of SbCAB2 (39.2%), and no inhibition was observed at 10 μg, with PO activity even surpassing that of the control group. Additional control experiments employing a human placental cathepsin B standard at concentrations of 0.625 μg, 2.5 μg, and 10 μg demonstrated no inhibitory effects on PO activity in *T. molitor* pupal hemolymph (Figure 9D–F). In summary, the results indicate that low doses of SbCAB2 from *S. bifidus* have an inhibitory effect on PO activity in the hemolymph of *T. molitor*, while high doses appear to have a promoting effect on this activity.

## 4. Discussion

### 4.1. Cathepsin Gene Repertoire and Evolution in Hemiptera

The cathepsin gene family was identified across 47 species of Hemiptera, with cathepsins B, L, and D being the predominant types, resembling those present in *B. mori*, *Tribolium castaneum*, and *T. molitor* [11,26,83]. Cathepsins B and L constituted more than 80% of the total, while cathepsins K, W, H, R, and S collectively represented less than 1% (Figure 1). In hemipteran and coleopteran species, the augmented utilization of lysosomal cysteine peptidases as primary digestive enzymes is linked to a notable expansion in the gene families cathepsin B and L [11,84,85]. This expansion was most prominent in aphids and whiteflies. For instance, in *Rhodnius prolixus*, the prominently expressed cathepsin B in the anterior midgut displays both exopeptidase and endopeptidase activities, facilitating hemoglobin degradation in the blood by initially breaking down the protein into short peptide chains through its exopeptidase function, which are subsequently further hydrolyzed by cathepsin L [25]. Likewise, *Dysdercus peruvianus DpCPL5*, highly expressed in the anterior midgut, undergoes a hydrophobic modification at the S2 subsite, diminishing substrate specificity and thus enabling the breakdown of plant proteins abundant in proline and aromatic residues [25]. Thus, this distribution pattern likely reflects the evolutionary adaptation of ancestral Hemiptera to low-protein plant sap, resulting in the reduction in digestive serine proteases (SPs) and the expansion of cathepsins B and L to fulfill protein hydrolysis demands [18,84,86,87]. In Hemiptera, cathepsins D (aspartic proteases) displayed suborder-specific evolutionary differentiation, with the suborder Heteroptera harboring a notably higher number of cathepsin D genes compared to the infraorders Sternorrhyncha and Auchenorrhyncha. The prevalence of cathepsin D genes in the suborder Heteroptera may be linked to selective pressures associated with specific metabolic needs related to feeding habits [18,88]. For instance, in the hematophagous *R. prolixus* A1 family, cathepsins D experienced gene expansion to aid in hemoglobin degradation within the acidic midgut environment during blood meals [88]. Similarly, the herbivorous *D. peruvianus* midgut exhibited numerous cathepsin D genes, potentially facilitating the supplementation of proteolytic activities of cathepsins L and aiding in the digestion of host plant cysteine protease inhibitors [89]. The suborder Heteroptera, encompassing diverse predatory, hematophagous, and herbivorous species, faces varied dietary sources (such as prey tissues, animal blood, and plants) and nutritional constraints, thereby propelling the expansion of cathepsins D within this taxonomic group.

In Hemiptera, the duplication and expansion of cathepsin genes represent the molecular basis for the adaptation of this group to diverse environments and hosts, driving species differentiation and niche expansion. This phenomenon is particularly typical in aphids and whiteflies. Cathepsin B achieves multi-level adaptation through gene expansion and functional differentiation. The pea aphid, *Acyrthosiphon pisum*, has adapted to the specific nutritional niche of phloem sap through large-scale expansion of this gene family, high intestinal expression, and positive selection of certain genes. Social aphids have evolved soldier-specific toxic cathepsin B to defend against natural enemies. The green peach aphid, *Myzus persicae*, and cotton aphid, *Aphis gossypii*, secrete cathepsins to regulate plant defense responses and promote host colonization [84,90,91]. The significant expansion of the cathepsin family in whiteflies (e.g., formation of lineage-specific tandem repeat gene clusters) facilitates adaptation to environmental stress. Cathepsin B and F in *B*. *tabaci* are highly expressed during viral co-infection or host switching, regulating virus acquisition and transmission. The extensively expanded cathepsin genes in this species are significantly upregulated after acephate treatment, cooperating with detoxification genes to participate in insecticide resistance [92,93,94]. These findings suggest that gene expansion, expression regulation, and neofunctionalization of cathepsins in Hemiptera represent key molecular mechanisms underlying their adaptive evolution and ecological diversification. It is noteworthy that hemipteran insects such as aphids, whiteflies, and scale insects possess unique cathepsin families—including cathepsin K, W, H, R, and S—which are relatively rare in other insect groups. Although the specific functions of these cathepsin families have not been systematically elucidated, their presence is considered closely associated with the unique ecological adaptations, host utilization, and co-evolution with plants in these insects. They may be involved in various physiological processes such as digestion, detoxification, immune regulation, or interactions with host plants [92,95].

### 4.2. Characteristics of S. bifidus Cathepsin Genes

Sequence analysis has shown that a majority of the cathepsins identified in *S. bifidus* contain varying numbers of N-glycosylation sites and signal peptides. These glycosylation sites have been speculated to regulate the lysosomal transport of cathepsin [96]. The presence of a signal peptide determines the secretion of cathepsin through the endoplasmic reticulum [97]. As such, it is suggested that these cathepsins utilize their glycosylation sites to facilitate secretion from the endoplasmic reticulum, subsequently becoming secreted proteins. Enzymatically active site residues, such as cysteine, arginine, glutamine, and aspartic acid, located in the mature peptide region of cathepsins, are crucial for their enzymatic activity [98]. In *S. bifidus*, three conserved enzyme catalytic sites, including cysteine, arginine, and glutamine, are found in cathepsins B and L. Meanwhile, cathepsins D contains two preserved aspartic acid residues. These findings suggest that cathepsins have the potential to display enzymatic activity.

The ERF/YNIN and GNFD motifs consistently appear in the cathepsin L subfamily, whereas the DTGS and DTGT motifs are conserved in the cathepsin D subfamily [13,99,100,101,102,103]. The ERF/YNIN motif is essential for the formation of the α-helix and exhibits a significant function in specific inhibition [100]. The DTGS and DTGT motifs are associated with two conserved enzyme active site residues [103]. These motifs have been correspondingly preserved among the *S. bifidus* cathepsins L and D. In cathepsins B, an occluding loop is featured as a fragment that is roughly 20 amino acid residues long [104,105,106]. This loop is bordered by two conserved cysteine residues on each side, and contains two intermediate histidine residues that determine whether or not cathepsin B exhibits exopeptidase activity [104,105,107]. In *S. bifidus*, three cathepsins B, namely SbCAB2, SbCAB3, and SbCAB5, possess this intact occluding loop, implying their exopeptidase activity. Contrastingly, both SbCAB1 and SbCAB4 lack the adequate histidine residues in this region, SbCAB1 is missing one, and SbCAB4 is missing both, suggesting that both SbCAB1 and SbCAB4 likely lack exopeptidase activity.

In C1 peptidases, the inhibitor_I29 domain functions as a propeptide, preventing substrate access to the active site [13]. The zymogen is activated when this N-terminal inhibitor domain is removed, either through interaction with another peptidase or via autocatalytic cleavage [108]. Therefore, it has crucial involvement in the activation of cathepsins L, O, and F in *S. bifidus*. As a family of cysteine protease inhibitors, cystatins imply an antiprotease function for the cystatin-like domain [109,110]. The existence of four such domains in the N-terminal section of SbCAF might be integral to the regulatory mechanism of this cathepsin’s physiological function. Additionally, the somatomedin_B-like domain is a novel domain identified for the first time in cathepsins in this study, located at the N-terminal region of SbCAB1. This domain is characterized by the presence of eight cysteine residues, forming four disulfide bonds [111]. Previous studies have shown that this domain, found in vitronectin, is involved in homodimerization as well as in mediating the binding of other proteins to cell surfaces and extracellular matrices [112,113,114]. These findings suggest that the somatomedin_B-like domain plays a role in the structure, stability, and function of SbCAB1.

### 4.3. Candidate Functions of S. bifidus Cathepsins

The gene functions are intimately linked to their specific expression patterns in various tissues. Using RNA-seq data, the expression patterns of cathepsin genes identified in *S. bifidus* were profiled in multiple tissues across different life stages. Genes with high expression levels in the salivary venom gland were further confirmed using qPCR. This echoed their expression patterns in RNA-seq profiling and endorsed the reliability of the results obtained through RNA-seq profiling. It was observed that most of these genes were expressed at the egg stage, highlighting their importance in embryonic development. It has been found in several insects, such as mosquitoes, blood-sucking kissing bugs, Asian citrus psyllids, as well as ticks, that certain cathepsins produced in the ovaries or developing eggs are involved in yolk protein degradation [115,116,117,118]. These cathepsins are essential for reproduction and embryonic development. As expected, apart from SbCAB2, SbCAB5, SbCAD5, SbCAL6, and SbCAO genes, all other identified cathepsin genes exhibit abundant expression in the guts at all nymph stages and in adults. Given the pivotal function of the insect gut in digestion, these cathepsins play a significant role in the degradation and hydrolysis of ingested food, attributed to their proteolytic activity [18,24]. It is intriguing that the majority of cathepsin genes, while highly expressed in the gut, show relatively low expression levels in the AG. The AG, one of the venom glands in *S. bifidus* and other bugs, is known to have a connection to the gut [43]. Hence, it can be proposed that these cathepsins, synthesized in the AG, might be transferred to the gut where they perform digestive functions. It remains to be confirmed whether these cathepsins can be injected into the prey’s body to aid in extra-oral digestion or other processes. In regard to the other two venom glands, PMG and AMG, *SbCAB1* and *SbCAB2* were highly expressed within them. Specifically, *SbCAB2* was found to be exceptionally expressed within the PMG. This suggests that the proteins these genes encode are venom components corresponding to these glands. The venoms from the AMG, PMG, and AG have been observed to play divergent roles, ranging from causing paralysis and tissue lysis, demonstrating insecticidal activities against prey, and inhibiting the melanization of prey’s hemolymph [39,41,43]. These cathepsins, as potential active venom components, might display various functions relating to their presence in specific venom glands. It should be noted that SbCAL6, SbCAB5, and SbCAO genes exhibited high levels of expression exclusively in the residual body, nymph or adult deprived of gut and salivary venom apparatus. This points to their significant roles in tissues apart from the gut and salivary venom apparatus.

### 4.4. Functional Role of SbCAB2 in S. bifidus Venom

Enzyme activity assays demonstrated that the recombinant proteinase activity of SbCAB2 (0.91 U/mg protein) surpassed the cathepsin activity of AMG (0.32 U/mg protein), AG (0.51 U/mg protein), and gut extracts (0.11 U/mg protein). This indicates that the baculovirus-expressed SbCAB2 recombinant protein exhibits biological function with hydrolytic capability, which can be used for physiological investigations [119,120,121]. Inhibition of PO activity experiments affirmed the inhibitory effect of SbCAB2 on PO in the hemolymph of the yellow mealworm. Notably, varying doses of SbCAB2 recombinant protein demonstrated that low doses (2.5 μg to 5 μg) inhibited PO activity, while a high dose (10 μg) led to an increase in PO activity. PO serves as a pivotal regulatory factor in insect immune defense. External stimuli trigger the activation of the inactive precursor prophenoloxidase (PPO) into active PO via the serine proteinase activating enzyme (PPAE), initiating an irreversible activation cascade that catalyzes melanin production to combat invading pathogens [122,123,124,125,126]. Serine proteinase inhibitors function to negatively regulate PPO activation by irreversibly inhibiting serine proteinase activation, thereby preventing systemic activation of this protein hydrolysis cascade [127,128,129]. Injection of the cysteine proteinase ScathL recombinant protein from *Sarcophaga peregrina* into fifth-instar *Heliothis virescens* larvae induces hemolymph melanization in the larvae’s hemocoel [130]. The cysteine proteinase DcCathL identified in *Delia coarctata* can specifically degrade the serine proteinase inhibitor MbSpn1A of *Mamestra brassicae*, thus impeding the negative regulation of serine proteinase inhibitors on PO [131]. The cysteine proteinase SbCAB2 from venom of *S. bifidus* can enhance hemolymph melanization at high doses, akin to other insect cysteine proteinases that selectively degrade serine proteinase inhibitors to elevate PO activity and augment hemolymph melanization.

Parasitoid wasps employ serpins in their venom to suppress the hemolymph PO cascade reaction of hosts, delaying the immune response and facilitating the development of their offspring [82,132,133]. Similarly, SbCAB2, a significant constituent of the venom of *S. bifidus*, potentially exhibits analogous physiological functions to serpins found in parasitoid wasp venom. At lower concentrations, SbCAB2 from *S. bifidus* may interact with the PO catalytic site or obstruct the proenzyme activation pathway to hinder melanization in the prey, mirroring the strategy of the predatory bug to prolong the predation process. Conversely, higher concentrations of SbCAB2 could trigger excessive melanization by dismantling or disrupting melanization inhibitor factors, resulting in reduced insect mobility, tissue injury, and lethal melanization [134,135,136]. Furthermore, Wu et al. [43] have revealed that the crude venom of *S. bifidus* can rapidly terminate prey and induce hemolymph melanization in the prey tissues [43]. Elevated doses of SbCAB2 venom might be utilized to interfere with the melanization of the prey, thus enhancing the efficiency of predation. Despite evidence indicating the impact of SbCAB2 venom on hemolymph melanization in vitro studies, further research is essential to ascertain the precise role of SbCAB2 as a venom component during predation, clarifying its contribution to the intricate interplay between prey defense mechanisms and the various venom components involved in predation.

## 5. Conclusions

This study reveals that the major types of cathepsins in Hemiptera are cathepsin B, L, and D, with cathepsin D exhibiting suborder-specific evolutionary divergence. The number of cathepsin D genes in Heteroptera was significantly higher than that in Sternorrhyncha and Auchenorrhyncha. A total of 22 cathepsin genes belonging to five subfamilies (CAB, CAL, CAF, and CAO) were identified from the genome of the predatory stink bug *S. bifidus*. Notably, *SbCAB2* was highly expressed in the venom gland, suggesting its potential role as a venom component. The recombinantly expressed SbCAB2 protein was able to affect the activity of phenoloxidase in the hemolymph of *T. molitor*. These findings provide important insights into the evolution of cathepsins in Hemiptera and the venom function of predatory stink bugs.

## Figures and Tables

**Table 1:** Characterization and classification of cathepsin in *Sycanus bifidus*.

Gene Name	Proteinase Type	Signal Peptide	MW (kDa)	PI	N-Glycosylation Sites
SbCAB1	Cathepsin B	22–23	50.88	8.57	2 (NSTC, NGTF)
SbCAB2		20–21	37.32	6.82	2 (NTTE, NGTF)
SbCAB3		–	71.70	5.71	3 (NCTI, NATP, NISY)
SbCAB4		–	32.68	8.88	4 (NKTL, NGTR, NITY, NGTD)
SbCAB5		16–17	37.28	6.66	3 (NTTW, NGTR, NGTR)
SbCAD1	Cathepsin D	23–24	35.58	5.04	0
SbCAD2		20–21	45.03	5.34	3 (NFTI, NQTF, NVST)
SbCAD3		17–18	42.07	5.55	2 (NLST, NQTF)
SbCAD4		16–17	41.59	4.95	3 (NGTE, NIST, NYTL)
SbCAD5		–	57.31	5.75	4 (NSST, NVSD, NETG, NVSF)
SbCAD6		22–23	22.23	9.54	1 (NQTF)
SbCAD7		18–19	48.49	5.85	2 (NISI, NKSS)
SbCAD8		–	39.87	5.24	6 (NGTV, NLTY, NCTS, NKTK, NTTG, NASE)
SbCAD9		20–21	46.31	8.86	6 (NGSG, NVSM, NVTN, NVTF, NFTD, NFTL)
SbCAL1	Cathepsin L	19–20	63.29	6.76	3 (NGTA, NMTC, NVTS)
SbCAL2		–	71.93	6.21	1 (NKSL)
SbCAL3		15–16	36.83	7.51	0
SbCAL4		19–20	35.31	5.08	1 (NLTQ)
SbCAL5		–	72.25	5.51	4 (NSST, NVSD, NETG, NVSF)
SbCAL6		16–17	37.62	5.23	0
SbCAF	Cathepsin F	26–27	89.94	6.99	5 (NATT, NITL, NRSE, NVSQ, NPTT)
SbCAO	Cathepsin O	25–26	39.75	8.21	4 (NKTY, NSSD, NKSN, NYSC)

Note: “– or 0” indicates that no signal peptide or N-glycosylation sites were predicted.

## Data Availability

The genome assembly data have been deposited in the NCBI BioProject database under accession number PRJNA1195740, and the RNA sequencing data from multiple tissues at different developmental stages have been submitted to the NCBI SRA database with accession numbers SRR24955820 to SRR24955862.

## Supplementary Materials

The following supporting information can be downloaded at: https://www.mdpi.com/article/10.3390/insects16111078/s1, Table S1. Identification of cathepsin reference sequences. Table S2. Cathepsin protein sequences of hemipteran species. Table S3. Cathepsin gene counts in hemipteran species. Table S4. Amino acid sequences of cathepsin from *Sycanus bifidus*. Table S5. qPCR primers used in this study. Figure S1. Multiple sequence comparison of *Sycanus bifidus* cathepsins L, F and O. Figure S2. Multiple sequence comparison of *Sycanus bifidus* cathepsins D. Figure S3. Multiple sequence comparison of *Sycanus bifidus* cathepsins B. Figure S4. Localization of *Sycanus bifidus* cathepsin genes on chromosomes.

## Abbreviations

Apis, *Acythosiphon pisum*; Acrc, *Aphis craccivora*; Agly, *Aphis glycines*; Agos, *Aphis gossypii*; Aluc, *Apolygus lucorum*; Asol, *Aulacorthum solani*; Btab, *Bemisia tabaci*; Clec, *Cimex lectularius*; Cced, *Cinara cedri*; Dvit, *Daktulosphaira vitifoliae*; Dcit, *Diaphorina citri*; Dnox, *Diuraphis noxia*; Elan, *Eriosoma lanigerum*; Fvir, *Ferrisia virgata*; Hhal, *Halyomorpha halys*; Hvit, *Homalodisca vitripennis*; Hcor, *Hormaphis cornu*; Lstr, *Laodelphax striatellus*; Mhir, *Maconellicoccus hirsutus*; Msac, *Melanaphis sacchari*; Mcer, *Myzus cerasi*; Mper, *Myzus persicae*; Nten, *Nesidiocoris tenuis*; Nlug, *Nilaparvata lugens*; Ofas, *Oncopeltus fasciatus*; Oins, *Orius insidiosus*; Olae, *Orius laevigatus*; Pven, *Pachypsylla venusta*; Pmar, *Paracoccus marginatus*; Pnig, *Pentalonia nigronervosa*; Psol, *Phenacoccus solenopsis*; Plon, *Pseudococcus longispinus*; Rpro, *Rhodnius prolixus*; Rmai, *Rhopalosiphum maidis*; Rpad, *Rhopalosiphum padi*; Rped, *Riptortus pedestris*; Sgrm, *Schizaphis graminum*; Schi, *Sclechtendalia chinensis*; Sflv, *Sipha flava*; Smis, *Sitobion miscanthi*; Sfur, *Sogatella furcifera*; Sc, *S. bifidus*; Tman, *Trabutina mannipara*; Tvap, *Trialeurodes vaporariorum*; Trub, *Triatoma rubrofasciata*; Tper, *Trionymus perrisii*.

## References

[1] Buttle M.D.J. (1997). Cathepsin B. J. Cell. Biochem..

[2] Willstätter R., Bamann E. (1929). Über die Proteasen der Magenschleimhaut. Erste Abhandlung über die Enzyme der Leukocyten. Biol. Chem..

[3] Turk D., Podobnik M., Kuhelj R., Dolinar M., Turk V. (1996). Crystal structures of human procathepsin B at 3.2 and 3.3 Angstroms resolution reveal an interaction motif between a papain-like cysteine protease and its propeptide. FEBS Lett..

[4] Conus S., Simon H.U. (2010). Cathepsins and their involvement in immune responses. Swiss Med. Wkly..

[5] Zavasnik-Bergant T., Turk B. (2010). Cysteine cathepsins in the immune response. Tissue Antigens.

[6] Turk B., Turk D., Salvesen G.S. (2002). Regulating cysteine protease activity: Essential role of protease inhibitors as guardians and regulators. Curr. Pharm. Des..

[7] Turk B., Turk D., Turk V. (2000). Lysosomal cysteine proteases: More than scavengers. Biochim. Biophys. Acta..

[8] Castino R., Pace D., Démoz M., Gargiulo M., Ariatta C., Raiteri E., Isidoro C. (2002). Lysosomal proteases as potential targets for the induction of apoptotic cell death in human neuroblastomas. Int. J. Cancer.

[9] Stoka V., Turk V., Turk B. (2007). Lysosomal cysteine cathepsins: Signaling pathways in apoptosis. Biol. Chem..

[10] Tran A.P., Silver J. (2021). Cathepsins in neuronal plasticity. Neural Regen. Res..

[11] Martynov A.G., Elpidina E.N., Perkin L., Oppert B. (2015). Functional analysis of C1 family cysteine peptidases in the larval gut of *Tenebrio molitor* and *Tribolium castaneum*. BMC Genom..

[12] Schmitz J., Gilberg E., Löser R., Bajorath J., Bartz U., Gütschow M. (2019). Cathepsin B: Active site mapping with peptidic substrates and inhibitors. Bioorg. Med. Chem..

[13] Wiederanders B., Kaulmann G., Schilling K. (2003). Functions of propeptide parts in cysteine proteases. Curr. Protein Pept. Sci..

[14] Correa K.C.S., Moreira A.C., Ibrahim A.G.A.E.-R., de Jesus H.C.R., Micocci K.C., Kock F.V.C., Bueno O.C., Venâncio T., Henrique-Silva F., Souza D.H.F. (2023). Identification and characterization of a recombinant cysteine peptidase (AsCathL) from leaf-cutting ant *Atta sexdens* Linnaeus, 1758 (Hymenoptera, Formicidae). Protein Expr. Purif..

[15] Greenberg B., Paretsky D. (1955). Proteolytic enzymes in the house fly, *Musca domestica* (L.). Ann. Entomol. Soc. Am..

[16] Gureeva T.A., Timoshenko O.S., Kugaevskaya E.V., Solovyova N.I. (2021). Cysteine cathepsins: Structure, physiological functions and their role in carcinogenesis. Biomed. Khim..

[17] Houseman J.G., Downe A.E.R. (1981). Endoproteinase activity in the posterior midgut of *Rhodnius prolixus* Stal (Hemiptera: Reduviidae). Insect Biochem..

[18] Terra W.R., Dias R.O., Ferreira C. (2019). Recruited lysosomal enzymes as major digestive enzymes in insects. Biochem. Soc. Trans..

[19] Saikhedkar N., Summanwar A., Joshi R., Giri A. (2015). Cathepsins of lepidopteran insects: Aspects and prospects. Insect Biochem. Mol. Biol..

[20] Di Y.Q., Han X.L., Kang X.L., Wang D., Chen C.H., Wang J.X., Zhao X.F. (2021). Autophagy triggers CTSD (cathepsin D) maturation and localization inside cells to promote apoptosis. Autophagy.

[21] Yang H., Zhang R., Zhang Y., Liu Q., Li Y., Gong J., Hou Y. (2020). Cathepsin-L is involved in degradation of fat body and programmed cell death in *Bombyx mori*. Gene.

[22] Pan G., Zhang K., Li C., Hu X., Kausar S., Gu H., Yang L., Cui H. (2021). A hemocyte-specific cathepsin L-like cysteine protease is involved in response to 20-hydroxyecdysone and microbial pathogens stimulation in silkworm, *Bombyx mori*. Mol. Immunol..

[23] Sun Y.X., Chen C., Xu W.J., Abbas M.N., Mu F.F., Ding W.J., Zhang H.J., Li J. (2021). Functions of *Bombyx mori* cathepsin L-like in innate immune response and anti-microbial autophagy. Dev. Comp. Immunol..

[24] Dvoryakova E.A., Vinokurov K.S., Tereshchenkova V.F., Dunaevsky Y.E., Belozersky M.A., Oppert B., Filippova I.Y., Elpidina E.N. (2022). Primary digestive cathepsins L of *Tribolium castaneum* larvae: Proteomic identification, properties, comparison with human lysosomal cathepsin L.. Insect Biochem. Mol. Biol..

[25] Pimentel A.C., Dias R.O., Bifano T.D., Genta F.A., Ferreira C., Terra W.R. (2020). Cathepsins L and B in *Dysdercus peruvianus*, *Rhodnius prolixus*, and *Mahanarva fimbriolata*. Looking for enzyme adaptations to digestion. Insect Biochem. Mol. Biol..

[26] Silva C.P., Dias R.O., Bernardes V., Barroso I.G., Cardoso C., Ferreira C., Terra W.R. (2022). Recruitment of lysosomal cathepsins B, L and D as digestive enzymes in Coleoptera. Insect Mol. Biol..

[27] Johnson K.P., Dietrich C.H., Friedrich F., Beutel R.G., Wipfler B., Peters R.S., Allen J.M., Petersen M., Donath A., Walden K.K.O. (2018). Phylogenomics and the evolution of hemipteroid insects. Proc. Natl. Acad. Sci. USA.

[28] Stork N.E. (2018). How many species of insects and other terrestrial arthropods are there on earth?. Annu. Rev. Entomol..

[29] Carver M., Gross G., Woodward T. (1994). Hemiptera (bugs, leafhoppers, cicadas, aphids, scale insects etc.). Insects of Australia.

[30] Panfilio K.A., Angelini D.R. (2018). By land, air, and sea: Hemipteran diversity through the genomic lens. Curr. Opin. Insect Sci..

[31] Terra W.R., Ferreira C. (2020). Evolutionary trends of digestion and absorption in the major insect orders. Arthropod Struct. Dev..

[32] Weirauch C., Schuh R.T. (2011). Systematics and evolution of Heteroptera: 25 years of progress. Annu. Rev. Entomol..

[33] Carvalho P., Martínez L.C., Cossolin J.F.S., Plata-Rueda A., Viteri Jumbo L.O., Fiaz M., Carvalho A.G., Zanuncio J.C., Serrão J.E. (2021). The salivary glands of *Brontocoris tabidus* (Heteroptera: Pentatomidae): Morphology and secretory cycle. Tissue Cell.

[34] Cantón P.E., Bonning B.C. (2020). Extraoral digestion: Outsourcing the role of the hemipteran midgut. Curr. Opin. Insect Sci..

[35] Cohen A.C. (1998). Solid-to-Liquid feeding: The inside(s) story of extra-oral digestion in predaceous Arthropoda. Am. Entomol..

[36] Yoon K.A., Kim W.J., Lee S., Yang H.S., Lee B.H., Lee S.H. (2022). Comparative analyses of the venom components in the salivary gland transcriptomes and saliva proteomes of some heteropteran insects. Insect Sci..

[37] Walker A.A., Weirauch C., Fry B.G., King G.F. (2016). Venoms of heteropteran insects: A treasure trove of diverse pharmacological toolkits. Toxins.

[38] Fry B.G., Roelants K., Champagne D.E., Scheib H., Tyndall J.D., King G.F., Nevalainen T.J., Norman J.A., Lewis R.J., Norton R.S. (2009). The toxicogenomic multiverse: Convergent recruitment of proteins into animal venoms. Annu. Rev. Genom. Hum. Genet..

[39] Walker A.A., Mayhew M.L., Jin J., Herzig V., Undheim E.A.B., Sombke A., Fry B.G., Meritt D.J., King G.F. (2018). The assassin bug *Pristhesancus plagipennis* produces two distinct venoms in separate gland lumens. Nat. Commun..

[40] Walker A.A., Hernández-Vargas M.J., Corzo G., Fry B.G., King G.F. (2018). Giant fish-killing water bug reveals ancient and dynamic venom evolution in Heteroptera. Cell Mol. Life Sci..

[41] Fischer M.L., Wielsch N., Heckel D.G., Vilcinskas A., Vogel H. (2020). Context-dependent venom deployment and protein composition in two assassin bugs. Ecol. Evol..

[42] Fischer M.L., Yepes Vivas S.A., Wielsch N., Kirsch R., Vilcinskas A., Vogel H. (2023). You are what you eat-ecological niche and microhabitat influence venom activity and composition in aquatic bugs. Proc. Biol. Sci..

[43] Wu C., Li L., Wang Y., Wei S., Zhu J. (2023). Morphological, functional, compositional and transcriptional constraints shape the distinct venom profiles of the assassin bug *Sycanus croceovittatus*. Int. J. Biol. Macromol..

[44] Fabricius J.C. (1787). Mantissa insectorum sistens species nuper detectas adjectis synonymis, observationibus, descriptionibus, emendationibus. Christ. Gottlieb Proft. Hafniae.

[45] Zhao P., Chen S., Liu Y., Wang J., Chen Z., Li H., Cai W. (2024). Review of the genus *Sycanus* Amyot & serville, 1843 (Heteroptera: Reduviidae: Harpactorinae), from China based on DNA barcoding and morphological evidence. Insects.

[46] Zhu J.Y., Yang P., Zhang Z., Wu G.X., Yang B. (2013). Transcriptomic immune response of *Tenebrio molitor* pupae to parasitization by *Scleroderma guani*. PLoS ONE.

[47] Chen C., Wu Y., Li J., Wang X., Zeng Z., Xu J., Liu Y., Feng J., Chen H., He Y. (2023). TBtools-II: A “one for all, all for one” bioinformatics platform for biological big-data mining. Mol. Plant.

[48] Mei Y., Jing D., Tang S., Chen X., Chen H., Duanmu H., Cong Y., Chen M., Ye X., Zhou H. (2022). InsectBase 2.0: A comprehensive gene resource for insects. Nucleic Acids Res..

[49] Li Y., Zhang B., Moran N.A. (2020). The aphid X chromosome is a dangerous place for functionally important genes: Diverse evolution of hemipteran genomes based on chromosome-level assemblies. Mol. Biol. Evol..

[50] Xie W., He C., Fei Z., Zhang Y. (2020). Chromosome-level genome assembly of the greenhouse whitefly (*Trialeurodes vaporariorum* Westwood). Mol. Ecol. Resour..

[51] Mathers T.C., Wouters R.H.M., Mugford S.T., Swarbreck D., van Oosterhout C., Hogenhout S.A. (2021). Chromosome-scale genome assemblies of aphids reveal extensively rearranged autosomes and long-term conservation of the X chromosome. Mol. Biol. Evol..

[52] Quan Q., Hu X., Pan B., Zeng B., Wu N., Fang G., Cao Y., Chen X., Li X., Huang Y. (2019). Draft genome of the cotton aphid *Aphis gossypii*. Insect Biochem. Mol. Biol..

[53] Nicholson S.J., Nickerson M.L., Dean M., Song Y., Hoyt P.R., Rhee H., Kim C., Puterka G.J. (2015). The genome of *Diuraphis noxia*, a global aphid pest of small grains. BMC Genom..

[54] Biello R., Singh A., Godfrey C.J., Fernández F.F., Mugford S.T., Powell G., Hogenhout S.A., Mathers T.C. (2021). A chromosome-level genome assembly of the woolly apple aphid, *Eriosoma lanigerum* Hausmann (Hemiptera: Aphididae). Mol. Ecol. Resour..

[55] Mathers T.C., Mugford S.T., Hogenhout S.A., Tripathi L. (2020). Genome sequence of the banana aphid, *Pentalonia nigronervosa* coquerel (Hemiptera: Aphididae) and its symbionts. G3 Genes Genomes Genet..

[56] Jiang X., Zhang Q., Qin Y., Yin H., Zhang S., Li Q., Zhang Y., Fan J., Chen J. (2019). A chromosome-level draft genome of the grain aphid *Sitobion miscanthi*. Gigascience.

[57] Kohli S., Gulati P., Narang A., Maini J., Shamsudheen K.V., Pandey R., Scaria V., Sivasubbu S., Brahmachari V. (2021). Genome and transcriptome analysis of the mealybug *Maconellicoccus hirsutus*: Correlation with its unique phenotypes. Genomics.

[58] Li M., Tong H., Wang S., Ye W., Li Z., Omar M.A.A., Ao Y., Ding S., Li Z., Wang Y. (2020). A chromosome-level genome assembly provides new insights into paternal genome elimination in the cotton mealybug *Phenacoccus solenopsis*. Mol. Ecol. Resour..

[59] Garber A.I., Kupper M., Laetsch D.R., Weldon S.R., Ladinsky M.S., Bjorkman P.J., McCutcheon J.P. (2021). The evolution of interdependence in a four-way mealybug symbiosis. Genome Biol. Evol..

[60] Ettinger C.L., Byrne F.J., Collin M.A., Carter-House D., Walling L.L., Atkinson P.W., Redak R.A., Stajich J.E. (2021). Improved draft reference genome for the Glassy-winged Sharpshooter (*Homalodisca vitripennis*), a vector for Pierce’s disease. G3 Genes Genomes Genet..

[61] Ma W., Xu L., Hua H., Chen M., Guo M., He K., Zhao J., Li F. (2021). Chromosomal-level genomes of three rice planthoppers provide new insights into sex chromosome evolution. Mol. Ecol. Resour..

[62] Liu Q., Guo Y., Zhang Y., Hu W., Li Y., Zhu D., Zhou Z., Wu J., Chen N., Zhou X.N. (2019). A chromosomal-level genome assembly for the insect vector for Chagas disease, *Triatoma rubrofasciata*. Gigascience.

[63] Rosenfeld J.A., Reeves D., Brugler M.R., Narechania A., Simon S., Durrett R., Foox J., Shianna K., Schatz M.C., Gandara J. (2016). Genome assembly and geospatial phylogenomics of the bed bug *Cimex lectularius*. Nat. Commun..

[64] Bai Y., Shi Z., Zhou W., Wang G., Shi X., He K., Li F., Zhu Z.R. (2022). Chromosome-level genome assembly of the mirid predator *Cyrtorhinus lividipennis* Reuter (Hemiptera: Miridae), an important natural enemy in the rice ecosystem. Mol. Ecol. Resour..

[65] Ferguson K.B., Visser S., Dalíková M., Provazníková I., Urbaneja A., Pérez-Hedo M., Marec F., Werren J.H., Zwaan B.J., Pannebakker B.A. (2021). Jekyll or Hyde? The genome (and more) of *Nesidiocoris tenuis*, a zoophytophagous predatory bug that is both a biological control agent and a pest. Insect Mol. Biol..

[66] Huang H.J., Ye Y.X., Ye Z.X., Yan X.T., Wang X., Wei Z.Y., Chen J.P., Li J.M., Sun Z.T., Zhang C.X. (2021). Chromosome-level genome assembly of the bean bug *Riptortus pedestris*. Mol. Ecol. Resour..

[67] Sparks M.E., Bansal R., Benoit J.B., Blackburn M.B., Chao H., Chen M., Cheng S., Childers C., Dinh H., Doddapaneni H.V. (2020). Brown marmorated stink bug, *Halyomorpha halys* (Stål), genome: Putative underpinnings of polyphagy, insecticide resistance potential and biology of a top worldwide pest. BMC Genom..

[68] Teufel F., Almagro Armenteros J.J., Johansen A.R., Gíslason M.H., Pihl S.I., Tsirigos K.D., Winther O., Brunak S., von Heijne G., Nielsen H. (2022). SignalP 6.0 predicts all five types of signal peptides using protein language models. Nat. Biotechnol..

[69] Letunic I., Khedkar S., Bork P. (2021). SMART: Recent updates, new developments and status in 2020. Nucleic Acids Res..

[70] Gupta R., Brunak S. (2002). Prediction of glycosylation across the human proteome and the correlation to protein function. Biocomputing.

[71] Chenna R., Sugawara H., Koike T., Lopez R., Gibson T.J., Higgins D.G., Thompson J.D. (2003). Multiple sequence alignment with the Clustal series of programs. Nucleic Acids Res..

[72] Minh B.Q., Schmidt H.A., Chernomor O., Schrempf D., Woodhams M.D., von Haeseler A., Lanfear R. (2020). IQ-TREE 2: New models and efficient methods for phylogenetic inference in the genomic era. Mol. Biol. Evol..

[73] Rambaut A. (2018). FigTree v1.4.4. Institute of Evolutionary Biology, University of Edinburgh. http://tree.bio.ed.ac.uk/software/figtree/.

[74] Su D.Y., Wang Y.Q., Li L., Wu G.X., Zhu J.Y. (2022). A protocol for dissecting the salivary gland from predatory bug—A case study in *Eocanthecona furcellata*. J. Environ. Entomol..

[75] Kim D., Paggi J.M., Park C., Bennett C., Salzberg S.L. (2019). Graph-based genome alignment and genotyping with HISAT2 and HISAT-genotype. Nat. Biotechnol..

[76] Love M.I., Huber W., Anders S. (2014). Moderated estimation of fold change and dispersion for RNA-seq data with DESeq2. Genome Biol..

[77] Simon P. (2003). Q-Gene: Processing quantitative real-time RT-PCR data. Bioinformatics.

[78] Sokolov B.P., Prockop D.J. (1994). A rapid and simple PCR-based method for isolation of cDNAs from differentially expressed genes. Nucleic Acids Res..

[79] Bradford M.M. (1976). A rapid and sensitive method for the quantitation of microgram quantities of protein utilizing the principle of protein-dye binding. Anal. Biochem..

[80] Saito H., Kurata S., Natori S. (1992). Purification and characterization of a hemocyte proteinase of *Sarcophaga*, possibly participating in elimination of foreign substances. Eur. J. Biochem..

[81] Dai Y., Jing T.Y., Wu J.C. (1989). Fluorescemine method applying to the determination of penicillin acylase activity. J. Hebei Univ..

[82] Yan Z., Fang Q., Liu Y., Xiao S., Yang L., Wang F., An C., Werren J.H., Ye G. (2017). A venom serpin splicing isoform of the endoparasitoid wasp *Pteromalus puparum* suppresses host prophenoloxidase cascade by forming complexes with host hemolymph proteinases. J. Biol. Chem..

[83] Li Y., Zhou X., Li Z., Li J., Chen S., Guo C., Hou Y., Zhao P. (2015). Identification and expression pattern of cathepsin family in silkworm (*Bombyx mori*). Chin. J. Biotechnol..

[84] Rispe C., Kutsukake M., Doublet V., Hudaverdian S., Legeai F., Simon J.C., Tagu D., Fukatsu T. (2008). Large gene family expansion and variable selective pressures for cathepsin B in aphids. Mol. Biol. Evol..

[85] Bansal R., Michel A. (2018). Expansion of cytochrome P450 and cathepsin genes in the generalist herbivore brown marmorated stink bug. BMC Genom..

[86] Houseman J.G., Downe A.E.R. (1983). Cathepsin D-like activity in the posterior midgut of hemipteran insects. Comp. Biochem. Phys. B..

[87] Cristofoletti P.T., Ribeiro A.F., Terra W.R. (2005). The cathepsin L-like proteinases from the midgut of *Tenebrio molitor* larvae: Sequence, properties, immunocytochemical localization and function. Insect Biochem. Mol. Biol..

[88] Henriques B.S., Gomes B., da Costa S.G., Moraes C.D.S., Mesquita R.D., Dillon V.M., Garcia E.S., Azambuja P., Dillon R.J., Genta F.A. (2017). Genome wide mapping of peptidases in *Rhodnius prolixus*: Identification of protease gene duplications, horizontally transferred proteases and analysis of peptidase a1 structures, with considerations on their role in the evolution of hematophagy in Triatominae. Front. Physiol..

[89] Pimentel A.C., Fuzita F.J., Palmisano G., Ferreira C., Terra W.R. (2017). Role of cathepsins D in the midgut of *Dysdercus peruvianus*. Comp. Biochem. Phys. B..

[90] Kutsukake M., Nikoh N., Shibao H., Rispe C., Simon J.C., Fukatsu T. (2008). Evolution of soldier-specific venomous protease in social aphids. Mol. Biol. Evol..

[91] Guo H., Zhang Y., Tong J., Ge P., Wang Q., Zhao Z., Zhu-Salzman K., Hogenhout S.A., Ge F., Sun Y. (2020). An aphid-secreted salivary protease activates plant defense in phloem. Curr. Biol..

[92] Chen W., Hasegawa D.K., Kaur N., Kliot A., Pinheiro P.V., Luan J., Stensmyr M.C., Zheng Y., Liu W., Sun H. (2016). The draft genome of whitefly *Bemisia tabaci* MEAM1, a global crop pest, provides novel insights into virus transmission, host adaptation, and insecticide resistance. BMC Biol..

[93] Lu D.Y., Liao J.Y., Fajar A., Chen J.B., Wei Y., Zhang Z.H., Zhang Z., Zheng L.M., Tan X.Q., Zhou X.G. (2023). Co-infection of TYLCV and ToCV increases cathepsin B and promotes ToCV transmission by *Bemisia tabaci* MED. Front. Microbiol..

[94] Shi X., Wang P., Shi C., Luo R., Zhang D., Zhang Z., Gao Y., Peng J., Preisser E.L., Liu Y. (2025). Cathepsin F alters viral acquisition, retention, and transmission of TYLCV and ToCV by *Bemisia tabaci* MED. J. Econ. Entomol..

[95] Mathers T.C., Chen Y., Kaithakottil G., Legeai F., Mugford S.T., Baa-Puyoulet P., Bretaudeau A., Clavijo B., Colella S., Collin O. (2017). Rapid transcriptional plasticity of duplicated gene clusters enables a clonally reproducing aphid to colonise diverse plant species. Genome Biol..

[96] Kim J.W., Cho J.Y., Kim J., Kim D.G., Nam B.H., Kim Y.O., An C.M., Kim B.S., Park J.Y., Kong H.J. (2020). First report of cathepsin E in a teleost (Korean rose bitterling, *Rhodeus uyekii*): Molecular characterisation and tissue distribution. Dev. Comp. Immunol..

[97] Wang B., Shi G.P., Yao P.M., Li Z., Chapman H.A., Brömme D. (1998). Human cathepsin F. Molecular cloning, functional expression, tissue localization, and enzymatic characterization. J. Biol. Chem..

[98] Rawlings N.D., Morton F.R. (2008). The MEROPS batch BLAST: A tool to detect peptidases and their non-peptidase homologues in a genome. Biochimie.

[99] Davies D.R. (1990). The structure and function of the aspartic proteinases. Annu. Rev. Biophys. Biophys. Chem..

[100] Guo Y.L., Kurz U., Schultz J.E., Lim C.C., Wiederanders B., Schilling K. (2000). The alpha1/2 helical backbone of the prodomains defines the intrinsic inhibitory specificity in the cathepsin L-like cysteine protease subfamily. FEBS Lett..

[101] Dunn B.M. (2002). Structure and mechanism of the pepsin-like family of aspartic peptidases. Chem. Rev..

[102] Pandey K.C., Barkan D.T., Sali A., Rosenthal P.J. (2009). Regulatory elements within the prodomain of falcipain-2, a cysteine protease of the malaria parasite *Plasmodium falciparum*. PLoS ONE.

[103] Guan Y., Yang X., Zhao R., Li B., Yang Z., Gao M., Cao X., Jiang C. (2022). Characteristics of cathepsin members and expression responses to poly I:C challenge in Pacific cod (*Gadus macrocephalus*). Fish. Shellfish. Immunol..

[104] Musil D., Zucic D., Turk D., Engh R.A., Mayr I., Huber R., Popovic T., Turk V., Towatari T., Katunuma N. (1991). The refined 2.15 A X-ray crystal structure of human liver cathepsin B: The structural basis for its specificity. Embo J..

[105] Illy C., Quraishi O., Wang J., Purisima E., Vernet T., Mort J.S. (1997). Role of the occluding loop in cathepsin B activity. J. Biol. Chem..

[106] Lambeth T.R., Dai Z., Zhang Y., Julian R.R. (2021). A two-trick pony: Lysosomal protease cathepsin B possesses surprising ligase activity. RSC Chem. Biol..

[107] Villalobo E., Moch C., Fryd-Versavel G., Fleury-Aubusson A., Morin L. (2003). Cysteine proteases and cell differentiation: Excystment of the ciliated protist *Sterkiella histriomuscorum*. Eukaryot. Cell.

[108] Groves M.R., Taylor M.A., Scott M., Cummings N.J., Pickersgill R.W., Jenkins J.A. (1996). The prosequence of procaricain forms an alpha-helical domain that prevents access to the substrate-binding cleft. Structure.

[109] Turk V., Bode W. (1991). The cystatins: Protein inhibitors of cysteine proteinases. FEBS Lett..

[110] Buša M., Matoušková Z., Bartošová-Sojková P., Pachl P., Řezáčová P., Eichenberger R.M., Deplazes P., Horn M., Štefanić S., Mareš M. (2023). An evolutionary molecular adaptation of an unusual stefin from the liver fluke *Fasciola hepatica* redefines the cystatin superfamily. J. Biol. Chem..

[111] Zhou A. (2007). Functional structure of the somatomedin B domain of vitronectin. Protein Sci..

[112] Schar C.R., Jensen J.K., Christensen A., Blouse G.E., Andreasen P.A., Peterson C.B. (2008). Characterization of a site on PAI-1 that binds to vitronectin outside of the somatomedin B domain. J. Biol. Chem..

[113] Chu Y., Bucci J.C., Peterson C.B. (2020). Identification of a PAI-1-binding site within an intrinsically disordered region of vitronectin. Protein Sci..

[114] Li T., Hao L., Li J., Du C., Wang Y. (2020). Insight into vitronectin structural evolution on material surface chemistries: The mediation for cell adhesion. Bioact. Mater..

[115] Fagotto F. (1990). Yolk degradation in tick eggs: I. Occurrence of a cathepsin L-like acid proteinase in yolk spheres. Arch. Insect Biochem. Physiol..

[116] Uchida K., Ohmori D., Ueno T., Nishizuka M., Eshita Y., Fukunaga A., Kominami E. (2001). Preoviposition activation of cathepsin-like proteinases in degenerating ovarian follicles of the mosquito *Culex pipiens* pallens. Dev. Biol..

[117] Zhao X.F., An X.M., Wang J.X., Dong D.J., Du X.J., Sueda S., Kondo H. (2005). Expression of the Helicoverpa cathepsin B-like proteinase during embryonic development. Arch. Insect Biochem. Physiol..

[118] de Almeida E., Dittz U., Pereira J., Walter-Nuno A.B., Paiva-Silva G.O., Lacerda-Abreu M.A., Meyer-Fernandes J.R., Ramos I. (2023). Functional characterization of maternally accumulated hydrolases in the mature oocytes of the vector *Rhodnius prolixus* reveals a new protein phosphatase essential for the activation of the yolk mobilization and embryo development. Front. Physiol..

[119] Götz B., Felleisen R., Shaw E., Klinkert M.Q. (1992). Expression of an active cathepsin B-like protein Sm31 from *Schistosoma mansoni* in insect cells. Trop. Med. Parasitol..

[120] Baek J.H., Lee S.H. (2014). Differential gene expression profiles in the salivary gland of *Orius laevigatus*. J. Asia-Pac. Entomol..

[121] Walker A.A., Madio B., Jin J., Undheim E.A., Fry B.G., King G.F. (2017). Melt with this kiss: Paralyzing and liquefying venom of the assassin bug *Pristhesancus plagipennis* (Hemiptera: Reduviidae). Mol. Cell Proteom..

[122] Ashida M., Yoshida H. (1988). Limited proteolysis of prophenoloxidase during activation by microbial products in insect plasma and effect of phenoloxidase on electrophoretic mobilities of plasma proteins. Insect Biochem..

[123] Kanost M.R., Jiang H. (2015). Clip-domain serine proteases as immune factors in insect hemolymph. Curr. Opin. Insect Sci..

[124] Whitten M.M.A., Coates C.J. (2017). Re-evaluation of insect melanogenesis research: Views from the dark side. Pigment. Cell Melanoma Res..

[125] Marieshwari B.N., Bhuvaragavan S., Sruthi K., Mullainadhan P., Janarthanan S. (2023). Insect phenoloxidase and its diverse roles: Melanogenesis and beyond. J. Comp. Physiol. B..

[126] Wang Y.H., Chang M.M., Wang X.L., Zheng A.H., Zou Z. (2018). The immune strategies of mosquito *Aedes aegypti* against microbial infection. Dev. Comp. Immunol..

[127] Janciauskiene S. (2001). Conformational properties of serine proteinase inhibitors (serpins) confer multiple pathophysiological roles. Biochim. Biophys. Acta..

[128] Zou Z., Jiang H. (2005). *Manduca sexta* serpin-6 regulates immune serine proteinases PAP-3 and HP8. cDNA cloning, protein expression, inhibition kinetics, and function elucidation. J. Biol. Chem..

[129] Bao J., Liu L., An Y., Ran M., Ni W., Chen J., Wei J., Li T., Pan G., Zhou Z. (2019). *Nosema bombycis* suppresses host hemolymph melanization through secreted serpin 6 inhibiting the prophenoloxidase activation cascade. J. Invertebr. Pathol..

[130] Li H., Tang H., Sivakumar S., Philip J., Harrison R.L., Gatehouse J.A., Bonning B.C. (2008). Insecticidal activity of a basement membrane-degrading protease against *Heliothis virescens* (Fabricius) and *Acyrthosiphon pisum* (Harris). J. Insect Physiol..

[131] Pyati P.S., Bell H.A., Fitches E., Price D.R., Gatehouse A.M., Gatehouse J.A. (2009). Cathepsin L-like cysteine proteinase (DcCathL) from *Delia coarctata* (wheat bulb fly): Basis of insecticidal activity. Insect Biochem. Mol. Biol..

[132] Colinet D., Dubuffet A., Cazes D., Moreau S., Drezen J.M., Poirié M. (2009). A serpin from the parasitoid wasp *Leptopilina boulardi* targets the *Drosophila* phenoloxidase cascade. Dev. Comp. Immunol..

[133] Zhou L., Wang R., Lin Z., Shi S., Chen C., Jiang H., Zou Z., Lu Z. (2023). Two venom serpins from the parasitoid wasp *Microplitis mediator* inhibit the host prophenoloxidase activation and antimicrobial peptide synthesis. Insect Biochem. Mol. Biol..

[134] Dubovskiy I.M., Whitten M.M., Kryukov V.Y., Yaroslavtseva O.N., Grizanova E.V., Greig C., Mukherjee K., Vilcinskas A., Mitkovets P.V., Glupov V.V. (2013). More than a colour change: Insect melanism, disease resistance and fecundity. Proc. Biol. Sci..

[135] Ahmed S., Kim Y. (2020). Prostaglandin catabolism in *Spodoptera exigua*, a lepidopteran insect. J. Exp. Biol..

[136] Mao Z., Wang B., Chen Y., Ying J., Wang H., Li J., Zhang C., Zhuo J. (2025). Musashi orchestrates melanism in *Laodelphax striatellus*. Insect Sci..

