# Supplementary material for: Comprehensive Analysis of Cathepsin Genes in Hemiptera: Functional Characterization of the Venomous Cathepsin B from Sycanus bifidus
Source: Insects. 2025 Oct 22;16(11):1078. doi: 10.3390/insects16111078 (PMC12653315; doi:10.3390/insects16111078)
Supplement: Supplementary file 1 [file insects-16-01078-s001.zip › Supplementary Files/Table S3.docx]

**Table S3.** Cathepsin gene counts in hemipteran species.

| **Toxonomy** | | **Feeding habit** | **Species** | **Cysteine proteases** | | | | | | | | | **Aspartic proteases** | **Total** |
| --- | --- | --- | --- | --- | --- | --- | --- | --- | --- | --- | --- | --- | --- | --- |
|  |  |  |  | **B** | **L** | **F** | **O** | **K** | **W** | **H** | **R** | **S** | **D** | **1744** |
| Sternorrhyncha | Psyllidae | Phytophagous | *Diaphorina citri* | 12 | 23 | 3 |  |  | 1 |  |  |  | 1 | 40 |
|  |  |  | *Pachypsylla venusta* | 14 | 18 |  | 1 |  |  |  |  |  | 1 | 33 |
|  | Aleyrodidae |  | *Bemisia tabaci* | 54 | 61 |  | 3 | 4 | 3 | 2 | 1 |  | 2 | 130 |
|  |  |  | *Trialeurodes vaporariorum* | 12 | 27 |  | 1 |  |  |  |  |  | 1 | 41 |
|  | Aphididae |  | *Acyrthosiphon pisum* | 24 | 1 | 1 | 1 | 1 |  |  |  |  | 2 | 30 |
|  |  |  | *Aphis craccivora* | 24 | 5 |  |  |  |  |  |  |  | 2 | 31 |
|  |  |  | *Aphis glycines* | 21 | 5 |  | 1 |  |  |  |  |  | 2 | 29 |
|  |  |  | *Aphis gossypii* | 17 | 4 | 1 | 2 |  |  |  |  |  | 3 | 27 |
|  |  |  | *Aulacorthum solani* | 23 | 7 |  | 2 |  |  |  |  |  | 3 | 35 |
|  |  |  | *Cinara cedri* | 17 | 11 | 1 |  |  |  |  |  |  | 4 | 33 |
|  |  |  | *Diuraphis noxia* | 16 | 2 | 1 | 1 |  |  |  |  |  | 3 | 23 |
|  |  |  | *Daktulosphaira vitifoliae* | 21 | 3 | 1 |  |  |  |  |  |  | 2 | 27 |
|  |  |  | *Eriosoma lanigerum* | 23 | 13 |  | 1 | 1 |  |  |  |  | 2 | 40 |
|  |  |  | *Hormaphis cornu* | 19 | 5 | 1 | 1 |  |  |  |  |  | 2 | 28 |
|  |  |  | *Melanaphis sacchari* | 18 | 3 | 1 | 1 |  |  |  |  |  | 2 | 25 |
|  |  |  | *Myzus cerasi* | 23 | 4 | 1 | 1 |  |  |  |  |  | 1 | 29 |
|  |  |  | *Myzus persicae* | 22 | 3 | 1 | 1 |  |  |  |  |  | 2 | 36 |
|  |  |  | *Pentalonia nigronervosa* | 20 | 4 | 1 | 1 |  |  |  |  |  | 2 | 28 |
|  |  |  | *Rhopalosiphum maidis* | 19 | 3 | 1 | 1 |  |  |  |  |  | 2 | 26 |
|  |  |  | *Rhopalosiphum padi* | 18 | 4 |  | 1 |  |  |  |  |  | 2 | 25 |
|  |  |  | *Schizaphis graminum* | 23 | 2 | 1 | 1 |  |  |  |  |  | 2 | 29 |
|  |  |  | *Schlechtendalia chinensis* | 18 | 5 | 1 | 1 |  |  |  |  |  | 2 | 27 |
|  |  |  | *Sipha flava* | 10 | 4 | 1 |  |  |  |  |  |  | 2 | 17 |
|  |  |  | *Sitobion miscanthi* | 2 |  |  |  |  |  |  |  |  | 1 | 3 |
|  | Coccoidea |  | *Ferrisia virgata* | 18 | 31 |  |  |  |  |  |  | 1 | 7 | 57 |
|  |  |  | *Maconellicoccus hirsutus* | 26 | 56 |  |  |  |  |  |  |  | 3 | 85 |
|  |  |  | *Paracoccus marginatus* | 15 | 11 | 1 |  |  |  |  |  |  | 2 | 29 |
|  |  |  | *Phenacoccus solenopsis* | 12 | 5 |  |  |  |  |  |  |  | 1 | 18 |
|  |  |  | *Pseudococcus longispinus* | 24 | 29 | 1 |  |  |  |  |  |  | 2 | 56 |
|  |  |  | *Trabutina mannipara* | 17 | 7 | 1 |  |  |  |  |  |  | 1 | 26 |
|  |  |  | *Trionymus perrisii* | 12 | 7 | 1 |  |  |  |  |  |  | 2 | 22 |
| Auchenorrhyncha,  Cercopoidea | Cicadellidae |  | *Homalodisca vitripennis* | 11 | 37 |  | 1 |  |  |  |  |  | 25 | 74 |
| Auchenorrhyncha,  Fulgoromorpha | Delphacidae |  | *Laodelphax striatellus* | 8 | 6 |  | 1 |  |  |  |  |  | 2 | 17 |
|  |  |  | *Nilaparvata lugens* | 6 | 10 |  | 1 |  |  |  |  |  | 3 | 20 |
|  |  |  | *Sogatella furcifera* | 9 | 10 |  |  |  |  |  |  |  | 1 | 20 |
| Heteroptera,  Cimicomorpha | Reduviidae | Hematophagous | *Rhodnius prolixus* | 5 | 10 | 1 | 1 |  |  |  |  |  | 20 | 37 |
|  |  |  | *Triatoma rubrofasciata* | 7 | 9 | 1 | 1 |  |  |  |  |  | 10 | 28 |
|  |  | Predatory | *Sycanus biffdus* | 5 | 6 | 1 | 1 |  |  |  |  |  | 9 | 22 |
|  | Cimicidae | Hematophagous | *Cimex lectularius* | 8 | 9 | 1 | 1 |  |  |  |  |  | 21 | 40 |
|  | Anthocoridae | Predatory | *Orius laevigatus* | 7 | 8 | 1 |  |  |  |  |  |  | 12 | 28 |
|  |  | Predatory | *Orius insidiosus* | 15 | 10 | 2 | 3 |  |  |  |  |  | 23 | 53 |
|  | Miridae | Phytophagous | *Apolygus lucorum* | 12 | 38 | 1 | 1 |  |  |  |  |  | 20 | 72 |
|  |  | Phytophagous | *Nesidiocoris tenuis* | 4 | 11 |  |  |  |  |  |  |  | 4 | 19 |
| Heteroptera，  Pentatomomorpha | Coreidae | Phytophagous | *Riptortus pedestris* | 20 | 53 | 1 | 1 |  |  |  |  |  | 19 | 94 |
|  | Lygaeidae | Phytophagous | *Oncopeltus fasciatus* | 12 | 17 | 1 | 2 | 0 |  |  |  |  | 14 | 46 |
|  | Pentatomidae | Phytophagous | *Halyomorpha halys* | 6 | 33 |  | 1 |  |  |  |  |  | 12 | 52 |
|  |  | Polyphagous | *Arma custos* | 4 | 20 |  |  |  |  |  |  |  | 13 | 37 |
