# Supplementary material for: Comprehensive Analysis of Cathepsin Genes in Hemiptera: Functional Characterization of the Venomous Cathepsin B from Sycanus bifidus
Source: Insects. 2025 Oct 22;16(11):1078. doi: 10.3390/insects16111078 (PMC12653315; doi:10.3390/insects16111078)
Supplement: Supplementary file 1 [file insects-16-01078-s001.zip › Supplementary Files/Table S4.docx]

**Table S4.** Amino acid sequences of cathepsin from *Sycanus biffdus*.

| **Gene ID** | **Gene name** | **Amino acid sequences** |
| --- | --- | --- |
| Scr_Chr02_00062.1 | SbCAB1 | MLKVVIILMIGTEILLNNVVESIIGPYDIPDGFCRDRADPKCCESRDDYCDRDDRGTKCYCDNFCHRSGDCCVDVNSTCYGKEDIVVKKCNYNGRFYNNGESFSHYCNTCDCMERDTFMELRCTMYTCVVDDKVADEINQIQNEWVAGRPQQFLSFKLSDAMSRMLGTFRSRKKVADMLPKYIEVRPRSLPAFFDAREKWRGKITPPPYQRCGASWVFSTVSVASDRWNIMSNGTFTGRLSFQHLLSCNKRNRMGCQGGHLTKGWSWIHDFGLVKEECYKWTGDNSRCHLPKQKGATSVRCLDNGRRTLTKLYYTAPVNRISGNQYQIMKEIMDNGPVQATMDIFRDFFLYESGVYSCPSHYKRSQIISVLPQITQYPAYHSVRIVGWGEQHINGKLVKYWIVANSWGESWGEGGFFRIRRGINSCNIEEFILAPWIHQY* |
| Scr_Chr03_00322.1 | SbCAB2 | MVGNLATLLVAIAVISSSKCQEPDENLINEINNMGLTWKAGRNFEERGYVRRLLANSKPRPRQTLPIMSRFNADIVIPDRFDSRQEWPDCPTISHIQDQGACGACWAVAGAATFSDRVCIASKGKHKLHLSAHHVLTCCGVKMCGTIACDGGDDYRAWVFFRINGVTTGGDYNTTEGCQPYTVEPCEHHTEGTRPNCYTMPAPVIAKCQTECTNKDYGKPMQQDLFKVSKAYKVMSDVNQIKREILKHGPVQAGFTVYEDFNVYKSGIYRHSVGNKVGEHSVRILGWGWEGNQQYWLVANSWNYNWGENGTFRVAMGVNECGIEEAVHAGVPIIE* |
| Scr_Chr04_00630.1 | SbCAB3 | MAEDFRRKYYLMSLVIAHITCSHAKLVIKELFEMRLVTEGRSGETIWTIKKEARAFELPKRLIKLKFKLPESFDSRKQWPKCLTISEIRDQGSCGSCWAFGAVEAMSDRICIHSGSKLNIHLSAENLLSCCSYCGFGCIGGYPGAAWNYWQNVGIVTGGNYGSNEGCQPYSIEPCQHHIEGPRPNCTIEGYNATPSCKDSCQKDYKGSYKDDLFYGNISYSIDNNPVAIQIEIYENGPVEGAYLVYDDFLSYKEGVYQYVAGTILGGHAIKLIGWGVEKNVPYWLVTNSWNSDWGDNGFFKILRGSNECGIEDFIYAGIPNLDEKSLITPGISLNCVEPLESRHSLPRRRFSAYQVLKEYCMLCSCAISVTPTFPIALPGMPQSGTSTFNLNMKLLILAPLLVCGVLSASVPADPLSDEFIDYINSLHTTWKAGRNFDKNVPRKYLKSINGVHEGANAFQLPKREVSLDVNLPEEFDARKQWPNCPSISEIRDQGSCGSCWGCQPYSIEPCEHHTTGSRPQCSGEGDTPDCKRSCRKGKSSYSIESDAEAIQTEIYKNGPVEAAFTVYEDFVNYKEGVYQHVAGGQLGGHAIRILGWGVENNVPYWLVANSWNSDWGDNGFFKILRGNNECGIEDQISAGLPKV* |
| Scr_Chr06_00764.1 | SbCAB4 | MLFLLILNIVYIVGSDLVNVINDNHIGWEAKSTLLETEEFREDILPEITPGRFTLAPVKGVNAKNKTLPETYDWRNERKGCNGTRVDGGPCANSWAIAPALVMTYRSCNSGKNITYSSVRILSCCTGCGAGCYGGYPWRAFNYMKSEGVPSEICQPLVIRSELPIRAIPPFCRIKNCDNNIAYKQNKIKSFYQVENIKNEIYERGPVVSYMNLYKDFFYYKKGIYKHKFGQKVGKLSVILLGWGTDNGTDYWIGENTWGSQWGENGYFRIIRGKNHVNIEQYVVAGLL* |
| Scr_Chr07_00643.1 | SbCAB5 | MKSFTIIFMLIYVVLCEYPPLDPLSDEFIDYINSLNTTWKAGRNFHPKTSRQYLKTLMGVHEDASQHQLPMKARSAINIMPPKEFDSRTNWPDCPTISEIRDQGSCGSCWAFGAVEAMSDRICIHSRGKVSVRLSSEDLVSCCYSCGFGCSGGFPGAAWSYWVHRGIVTGGSFGTNQGCQPYKIEPCEHHINGTRKPCDKHMSRTPKCVKTCLPNYKKTYKQDLHYGKSAYQIENDEHSIQTDIMNYGPVEGTLTVFEDLLTYKEGVYKHVAGKALGGHAIRILGWGEENGTPYWIIANSWNTDWGDHGYFKILRGSDECGIESGISAGIPKD* |
| Scr_Chr01_00061.1 | SbCAD1 | MRSANLLHVFVILLSFLVVIISAQQSSVILHRRTKGLKSLSDFTKSLKQWKQHFLHYNTLKAKTGESFISLDYGKVKLFNSMNVEYFAEIFIGNPPQKFTVAVDTGSSDLWIPSKQCTTFNVACWFHSKYDHSKSITYKDTGETMNIAYLTGSMSGLVSQDEVRVCFNNKYTEDEKDDFGGELLFGGIDETKFNKSTLQYVPLTQQTYWQFALDGLKIGELEIDLDDAEAIADTGASLILGEEEIVNNFYRNVGAEIDSGGNAYVSEFFFWTKCVIGIAGMDLNGEQWILGDVFLGKFYTIFDGANSQIGFAERIH* |
| Scr_Chr01_00062.1 | SbCAD2 | MRYLVLFATILVLCFHEIYGINRVTLLKRNIGLRPSGEFLNNIKQWKHQFNEFQTLKQLPESKSRADAGRVTVKNYLNLNYYIPITIGNPPQNFEVGIDTGSSSLWIPSKHCSFFNIACWVHHTYNHDKSSTYIPVGDAIDFRYVTGHVKGYISKDEITVGNFTITNQTFGEATEEPGFTFVAAEYDGILGLAFPILSDTGLPVHYKMLFQSLVPEVLFSVYLNRNEKDNFGGEIIFGGYDEDKFNVSTLTYIGLSQNTFWQFKLDGLQIGELQIDLDHREAIADTGSTVIVGDKDIVENFYRIIGATIEGDGAYVDCDKIDQLPPLDFVIGNKKYRLEGKDYIIKVNVYIFWKRCYIGIIGMDLGSEPWILGDGFLGKFYTIFDGTNSRIGFAELK* |
| Scr_Chr01_00306.1 | SbCAD3 | MLLFTIFLISAFCGVLSGSGNLVRVPLTKIQSARRTFQEVGTAIEQLSLKYAGNAAGPFPEPLSNYLDAQYYGPITLGNPPQSFRVVFDTGSSNLWVPSKKCSKLNIACWVHRKYDSSKSKTYVPNGQKFAIAYGSGSLSGYLSEDNLSIGGIAVANQTFAEAINEPGMVFVAAKFDGILGLGYDTISVDNVPPPFYNMYQQGLVQNPVFSFYLNRDAAASIGGEIIFGGSDPDKYTGDFTYIDVNKKGYWQFSMDSILVSGKSFCKGGCQAIADTGTSLIAGPTEEVTAINQLIGGTPISGGEYMVACDLIPKLPTIDFVLGGKKFSLEGKDYILRISALGKTVCLSGFLGMDIPPPHGPLWILGDVFIGRFYTEFDLGNNRVGFAIAKE* |
| Scr_Chr01_00339.1 | SbCAD4 | MRTIILLTFISAISDAQISLKLTRWNKLFANIPLNPIVLQSIIRNGGGTRQSLTSHKNVYYTGLITLGTPPQEFNVNFDTGSTNLWIYSRECWWSAACWTHRNYKHDLSKTYEENGTEVRVVYGTGSMEGFLSNDILGLGNISTPVNFIEATKVPELADVFLQFDGIFGLGLQDRQIALPAFLSIIDQLPQPIFSVYFNRNAKNEKDNGGEIIFGGVDPSKFVKSTLTMHNVATDSTWAVPIDSVFVGDVLAVSCDEGCTGVIDTGTSFIITPEKPLNDIRNVIGINDGDGAVPCEDIKNLPPIKFLIGKKNYTLTSSQYVMKKTSLWMDSCYDSFYNMDLKGLWILGDVFLGHFYTIFDYENKQIGFADLVE* |
| Scr_Chr03_00400.1 | SbCAD5 | MEDAAQYYGDIGIGTPSQTFSVVFDTGSSELWVPSVECLSTVCGNHRRFNSSTSKTFVATDYPAFIQYGIGSIYGKLGADKLQIGELVIHNQQFIEALEQSKEPFLFAAMDGVMGLSLSSKNNVKSNVPLSNMIQQNLLEKPIFSFYLNRNVSDSFGGELMFGGVNNDLFISDTLHSVPLYEGIYWVIMIDSISIPKLSQTFCTEGCFGLVDTGTSLIVGPQKDVRSIFRTVGAKYKDGIGAYQTDRGKRLTGIPTGGFQEKVTKRWQHDEALPTRAPKQHTLKPSAVPQFPTLRKVPKTEQIEHPVITGVEKDEIERRESESETSEGYRGENDHVSEKQRPVTPEPGRKQSNEDEFRPIIRMSSTADYTGTLQERRQRQSVQPHVPPIAAGLLTSGDSSIEIYTDSEVSLWIIEDHKNHNETGERIRRFLRKLRLEMGWTVNIYWIKAHVGHYGNELADRLAKEASFDDELNVSFSKVPLSYVTKSANAQAMERWEKEWSKTENGQETK* |
| Scr_Chr03_00401.1 | SbCAD6 | MYSNSVLVALVLLVAALITSNAVLRIPLQKVHKAPRKYSEFVQTVQKSHSSLDNYLFLQKNGVKEVLKNNFNIGSTRIENQTFGEMTSLSRKPFRRAKFDGILGLGYPSIAVNHVTPPVPCEMTSVLPAITFTIGGRPYVLQQQDYILQMKDGDKLTCVVGFLSLPSLSQGTWILGDVFLGKFYTVFDFGENTVSFGKLK* |
| Scr_Chr03_00402.1 | SbCAD7 | MKFLAILLIVLPIILVSSTGVVKIQLHKRAPPTFEEHLSNRNLYQQALARFMVLPSYGGKETVELRNFVNSQYYGNISIGSPPQEFQVLFDTGSTLLWVPSVDCYSSACKTHKNFKNYLSSTYRPSSYQIKIVYGKGSMSGVMGRDTVQIGSLIVDNQQFAEAVEEPGSAFVSSKFDGIMGMAFPTRGSSIEPVFQTMYKQGVIKDNVFSFYLSRDPNGYLGGEMVLGGWNDFYFDSSKIDYIPLSKVDKWQFTIDFISGRGGSFEDSPGSFYLGGCEAIADTGTTMIIGPVQDISAIHKFIGAEESYGMGMVPCNKVDQLPSITFHINGKEYTLEGRDYVVNLADDYIMCITGFTGMNMASRPWILGDAFLGKFYTIFNVQDESVAFAPLNKSSRKDTKFVPQHPENKPYFPGNAPAVQVTFLTLFIANLAHWFL* |
| Scr_Chr03_00404.1 | SbCAD8 | MEKTIQARENSGNINGTVTALNLTYSHGFIGAISIGTPINDQVFKVLIDTESTVLWVPSSNCTSGVCRNHHVYSYQWSKTYESNDRFKTIQDGEDKMLGLGGKDVVQIGSVSLKSVPFYTVNDLPVDFLTKKTYDGGLGLSLPIKSDDETILDSMINQGALEWNLFSLYINKTKESKYTKYLGGELMLGGWNTKYFNPDDIEYIPLSKPGKWQFTIDRITSVENTTGVWCNSGCEATISTTFPLIQGPAKEIKAIYQYIGASIDLLPGIPLIKCNQIDQLPPIHFHINNKEYILESTDYTVQWNAEVSGGIAAELCLSAFVIAENQNDPWKIGYQFLSKYYTIFNASEKKFAFATLK* |
| Scr_Chr08_00790.1 | SbCAD9 | MFTQGLIFSALLLCCSYSTAYHHDEDYYEVNTYNSGNVIKIPLIRMESALQRMARENIHPRVIESFFMKAAKNGSGVPVPLFKFLDTEFYGEILIGHPGQKFKVVFDTAWTNTWVPSVLCPVIEVACILRNKYDASRSSTYVKDNRPFNVSMGSLQLKGKLSTDLFHVNQVNVTNVTFAEINEIPWILFFSKADGVVGLAFADFAVDGVTPLFYDMIKQGVVDQRIFSFYMNRDPASPKGGTIMFGGVEKRHYLGNFTDVKIIPKTGLWSFQIDRIFTSRKKSDQFCTSGCQAFADTSENTIRGPPDDIEMLNSIIGAQSFYFGRYIVNCNNVNTLPKVTFFIKNRNFTLKGQDYIQRLTWGPVTICLSSFKKSEIPNTWALGAAFLSRYYVRFDLQRMLIGFADARL* |
| Scr_Chr02_00446.1 | SbCAL1 | MIEFKNLVVLLGFFYVASGCRQDTPPVFSSTYSVRGVLNIPYAELREPFFAWYDSQNARSRIDYYGGTVKTYQISGMGPYGALLKIAPVTTEDQWNVDTCLQVNGTAQEKVKPQTILPDLQKFKCLGTDILNGVECELWKYVENVGEKTNKYTMWITWKTSPKTRRLARAEPVRYEMKGYNNLLGSHFDHYYLDYDLYSTEQPAPETFKVNTNMTCTNFPGPGRYHTATFNPLKEFVHNDDQHVDKEWEDFVKKHSKVYNKEQLEHHKRKEVFRQNLRFIHSQNRAKLGFNLAINHLADRTEDELRALRGRKYSTDNNGASPFPYSLGEMKKLSEDLPDQFDWRLYGAVTPVKDQSVCGSCWSFGTTGAVEGAYFVKTGHLIRLSQQALVDCSWGYGNNGCDGGEDFRSYQWIMKHGGLPLESDYGGYLGQDGYCHIDKVPLTAKITGYVNVTSNDENALRLALFKHGPISVAIDASHRTFSFYSNGVYYDPECKSRVDQLDHAVLAVGYGKMNGKDYWLIKNSWSNLWGNDGYVLMSAKDNNCGVMTSPTYVTF* |
| Scr_Chr10_00493.1 | SbCAL2 | MSWKHFKQLYRKKYPNPLEEHYRKQIFEENVNKIEEHNRAFANGQMSYTMKINEFSDMMSNEVKTLMNGFKMSANTKRNGQKYVPFNGKLPESVDWRQKGAVTPIKDQGQCGSCWAFSTTGSLEGQLFLKKGKLVSLSEQNLVDCSGDYGNNGCGGGLMDQAFQYIKDNKGIDTEQSYPYEAVDQKCRFKASKVGGTDKGYVDIPEGDEEALKNAVASVGPISVAIDAGHDSFQSYGGGVYNEPYCSTSSLDHGVLAVGYGTENGQDYWLVKNSWGTSWGENGYIKMARNKNNHCGIASMASYPLEMMKLIIFLSIFMAVSQAITFFDLVQEEWNLFKLQHKKSYSSDVEEMFRMKIFMENKRKVAKHNARYSQGLVKFKLGINHLADMLPYEFAKYNRYNKSLLATNEPKLIGATFIEPANVELPLHVDWREKGAVTPVKNQGQCGSCWSFSSTGALEGQHFRKTGKLVSLSEQNLIDCSKKYGNNGCNGGLMDYAFQYVKDNHGIDTEKSYPYEADDDKCRYKSRDSGATDNGFVDIPEGSEDKLKAAVATIGPVSVAIDASHESFQLYAEGVYYEPECSSQDLDHGVLVVGYGTTEEGEDYWLVKNSWGRRWGLQGYIKMARNKDNNCGIASSASYPLV* |
| Scr_Chr10_00494.1 | SbCAL3 | MKVIIALCIVGLSYAFPPNGVFPTEWMEFKLTHGKNYKNLFEEQIRMRVYMNNKKRIEEHNAKYEQGLVSYAMKMNHFGDLMPHEFKAVMNKLKFNINSKRNGIKYIPSNDKIPESVDWRQKGAVTPVKDQGQCGSCWAFSTTGSLEGQLFLKKGKLVSLSEQNLVDCSGDYGNNGCDGGLMDQAFQYIKDNKGIDTEQSYPYEAVDGECRFKSSNVGGTDKGYVDIKQGDENDLKNALATVGPISVAIDAGNESFQFYSKGIYNEPYCSSSSLDHGVLAVGYGSDNGQDYWLVKNSWGPKWGEQGYIKMIRNKNNRCGIATMASYPLV* |
| Scr_Chr10_00495.1 | SbCAL4 | MRELFIVFVTIFLLCRSHALLKDDWISFVETHGKVYDDPIEAAVRMSIFAVNKEMIDKHNVDYDLGKVSYKLKMNHFGDLRNLTQKSKSVRSFIPPANAQLPDFVDWRKTGAVTPIKDQGHCGSCWSFSATGALEGQYFLKTGKLLSLSEQNLMDCSTTYGNQGCDGGLPDLAFQYISDNHGIDSEDSYPYEAKDNLCRYNPLHSVSSDRGYIDLPIGDEHALKAAVATVGPISVAIDASSNDFKFYSEGIYDEPSCSSSDLDHAVLAIGYGTTETGDNYWLVKNSWGEDWGEQGFVKMPRNKNNRCGIATSAAYPLV* |
| Scr_Chr10_00496.1 | SbCAL5 | MPPTVQDIDSWMNNMQSQWQNFVETNNKQYKDSKEEEFRFHVYAYNTFKIKLHNMMYDFGKYSYKLKMNEYGDLTAQEFGLIKKGYFGLLTNETDFGVGGRYFYYTPKGPVNKTVDWRKLGAVTEVKNQGNCGSCWSFSTTGSLEGQHFLKTGKLVPLSEQNLIDCSYFNGNNGCNGGLMEDAFRYIKENGGIDAEKDYPYEGIDVATVNGFTTIPKGNETLLKYAVATVGPVSVGIDASSSSFQFYSEGVYNDLSCSSSKLDHGVLVVGYGTTEDGKDYWLVKNSWGPKWGDKGYIKMSRNKDNQCGIASYALFPLIIMKVALCLLFGIVAIQGAWVNNAPEDWMAFKANFGKVYKNSFEELFRMNVYRDNLRKIEEHNKKFENGEVTYSLKMNHFGDLMQHEFKAMMNRLKPSKKASNNKVFEATGVKVAETMDWRDQGAVTAIKDQGQCGSCWAFSTTGSLEGQLFLKNHKLVSVSEQQLVDCSGDYGNEGCNGGLMDSAFEYIKANGGIDTEASYPYEAEDDKCRFNKKNVAGTVIGFVDIKSGDENALKEAVGSIGPVSVAIDAGNLSFQFYSSGIYNEPWCSDSSLDHGVLAVGYGTDNNNDYWLVKNSWGTVWGMDGYIKMTRNKNNQCGIATQASYPLV* |
| Scr_Chr10_00498.1 | SbCAL6 | MQILIAFCILTRLISAIPTYTLQNIQEWKYFKETFGKSYEDPNVDLKRMEIYFRNKNFINNWNRLYQHGVKSHQLKMNHYSDMYPNEFRAYVNGFNNSIVNNDDNIKFVIPQELEIPESLDWRDNGAVTEVKDQSECGASWAFSATGAIESHHFIKTGQLILLSEQNLIDCSDDYGNNGCVSGNMENAFRYIKNAGGINPEVSYDYEAVKGICRFKREYAVNMNGFVNLPEGDEEALKVAVASIGPVSVAIDAGRLSFQFYGSGIYFDPECSTTNLNRGMLIVGYGTDIHGTDYWLMKNSCSTLWGEDGYIRIARNKLNHCGIASAASYPVM* |
| Scr_Chr05_00552.1 | SbCAF | MRMGSLKIFTFQFLIVLITLNSCVKADICAGCPIDHDLNDPEIKQQLKQVLSSKNEDAQIMKLLSAKTQVINGIKYDVSFQIQNSRTKEVKVCHTVYVSRPWLSKQLYIDEFNCDSVEHPKNARQLLGTVKDMGADEKDLVSHLADLAVKAIDDVDVDDSKKIVTEIVDAKKQLVNGIMYHLTLKVGTTSCNEDDLNTIPANCHLDKTVPQEICKIKMHRSFADISPLKARFVNSECDEIKNFLGFSVESDIVKEAADFAAERINEMSNSIYKQVLLQILGATSKAASDVQMDLTLELGNTECMKNKHDQPNCGIVPDNAEKMICTVSVLSEPWKKTNGKSYMKIATFKCGPFFRAKRSLAGGQSGLDINDKKVIDLKNYIEDELTSRSNSQHTKTVVKVLNATTQIVSGTMTRITVEVADTNCLKGENKEKSQCTATDQGHQICDLAIWEQPWLLKKEITQSECHSINNNHGSNGRQKRMADILALEGDFKTVQDFLGFMTREGKIYKDNVEMVRRFKIFRANMMKAAFYQKHEQGTAIYGATMFADLTEEEFKKYLGYKVNSNFKSKVERKMAVVPNITLPTEFDWRNYNAVTNVKNQGMCGSCWAFSVTGNIEGLWAIKNKQLLSLSEQELVDCDKYDKGCMGGEFDTAYQAIEDIGGLETETDYPYKGWGEKCHFNRSEVRVSITGAYNVSQNEEDMAKILVKNGPISIALNANAMQFYMGGVSHPLRFLCSPSNLDHGVLLVGFGVHRTRFTHKNLPYWLIKNSWGTRWGNKGYYMLYRGDGSCGVNQNPTTAEL* |
| Scr_Chr14_00007.1 | SbCAO | MLEWKSVCLTLGLIALCFVGIPIKMDNLRSSVQHLTFNSYLELYNKTYKPESDEYHIHFKHFQDGKKRWQHLNKQRNSSDLAYYGFTEFADISQEEFSLRYLKHHRHGHWNKSNDALSTKSETITLLKLTISGDNIPTKVDWRERGIVSRIKNQGLCGACWAFSIVSTAESMLALKTGQLKDLSVQEMIDCAGNGNLGCNGGDTCSLLEWLVNNKINIETEKEYPLVLKNEVCKLKRTLTGVRIAPNYSCRSYVGAENSLLSVIANHGPVTVAVNALTWQYYLGGVIRFNCDNSIQSLNHAVVIIGYDLTAKTPHYIVRNSWGTKFGESGYLKIAIGDNVCGIAHEISVFDVL* |
