# Supplementary material for: Comprehensive Analysis of Cathepsin Genes in Hemiptera: Functional Characterization of the Venomous Cathepsin B from Sycanus bifidus
Source: Insects. 2025 Oct 22;16(11):1078. doi: 10.3390/insects16111078 (PMC12653315; doi:10.3390/insects16111078)
Supplement: Supplementary file 1 [file insects-16-01078-s001.zip › Supplementary Files/Table S5.docx]

**Table S5.** qPCR primers used in this study.

| **Gene name** | **Forward primer sequence (5'-3')** | **Reverse primer sequence (5'-3')** |
| --- | --- | --- |
| *SbCAB1* | AATAACTCCACCTCCGTATC | GCTATTGTCTCCAGTCCAT |
| *SbCAB2* | GCGATGGAGGTGATGATTA | GGCATTGTATAGCAGTTTGG |
| *SbCAD2* | ATTGGTGCCACGATAGAG | ATCCATCGCCAAGAATCC |
| *SbCAD3* | CTATGGATCTGGAAGCCTAT | CCAAGACCAAGGATACCA |
| *SbCAD9* | GCATGTTGGGTTCATCAC | AAGCCAGGTTCTTCAGTAG |
| *SbCAL1* | CATCACCATTCCCATATTCC | CCATCACATCCGTTATTACC |
| *SbCAL2* | TGTGGTGGAGGACTTATG | CGATAGCAACAGAGATTGG |
| *SbCAF* | CAGAAGAGGAGTGGCAGATA | CATAGTAGCACCGTAGATTG |
| *SbRPS3A* | TTCTGGTGTTGAGGTTAGAG | AGCAAGTCCGCCAATTAG |
