# Supplementary figures and images for: Comprehensive Analysis of Cathepsin Genes in Hemiptera: Functional Characterization of the Venomous Cathepsin B from Sycanus bifidus
Source: Insects. 2025 Oct 22;16(11):1078. doi: 10.3390/insects16111078 (PMC12653315; doi:10.3390/insects16111078)

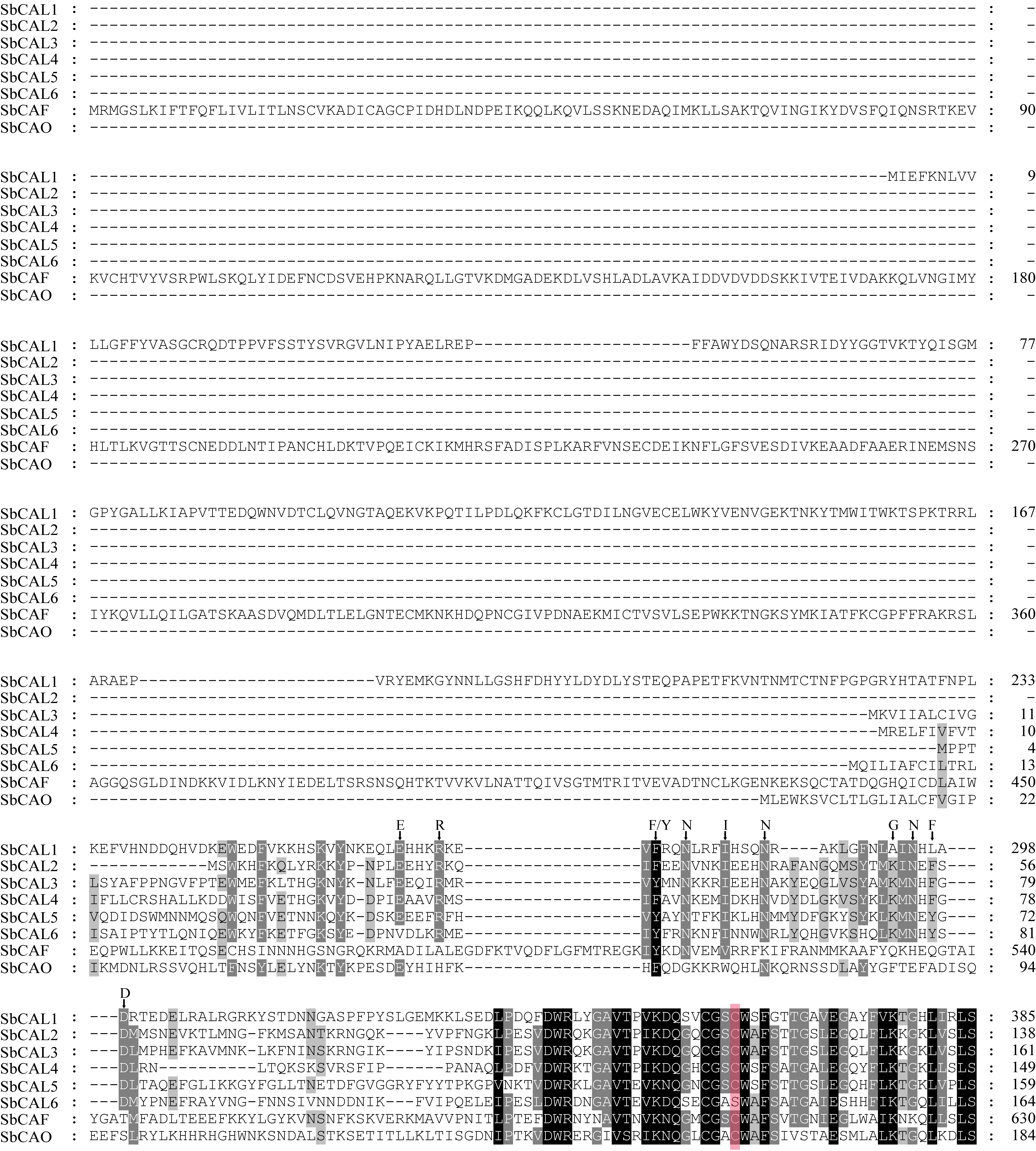


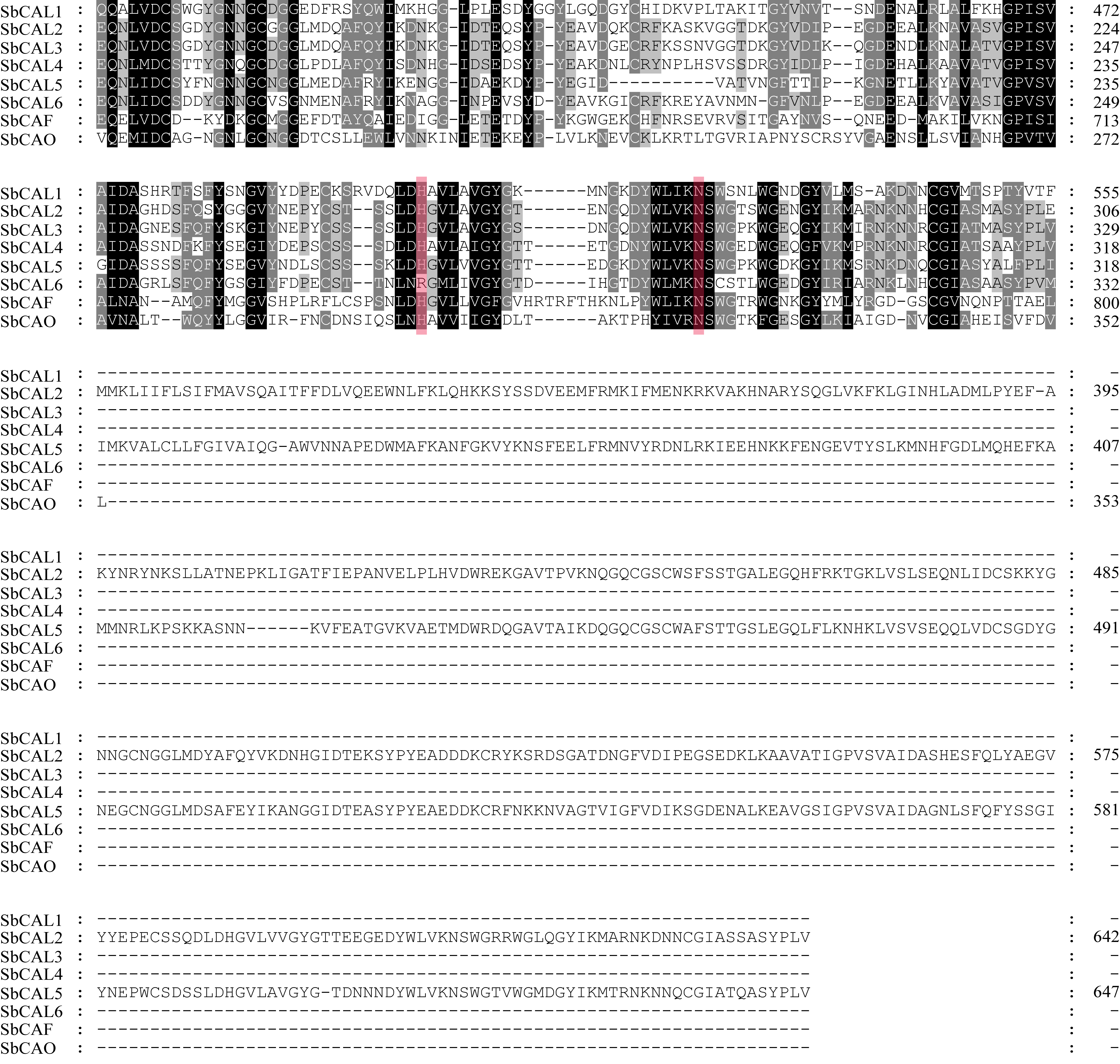

Supplement: Supplementary file 1 [file insects-16-01078-s001.zip › Supplementary Files/Figure S1.docx]

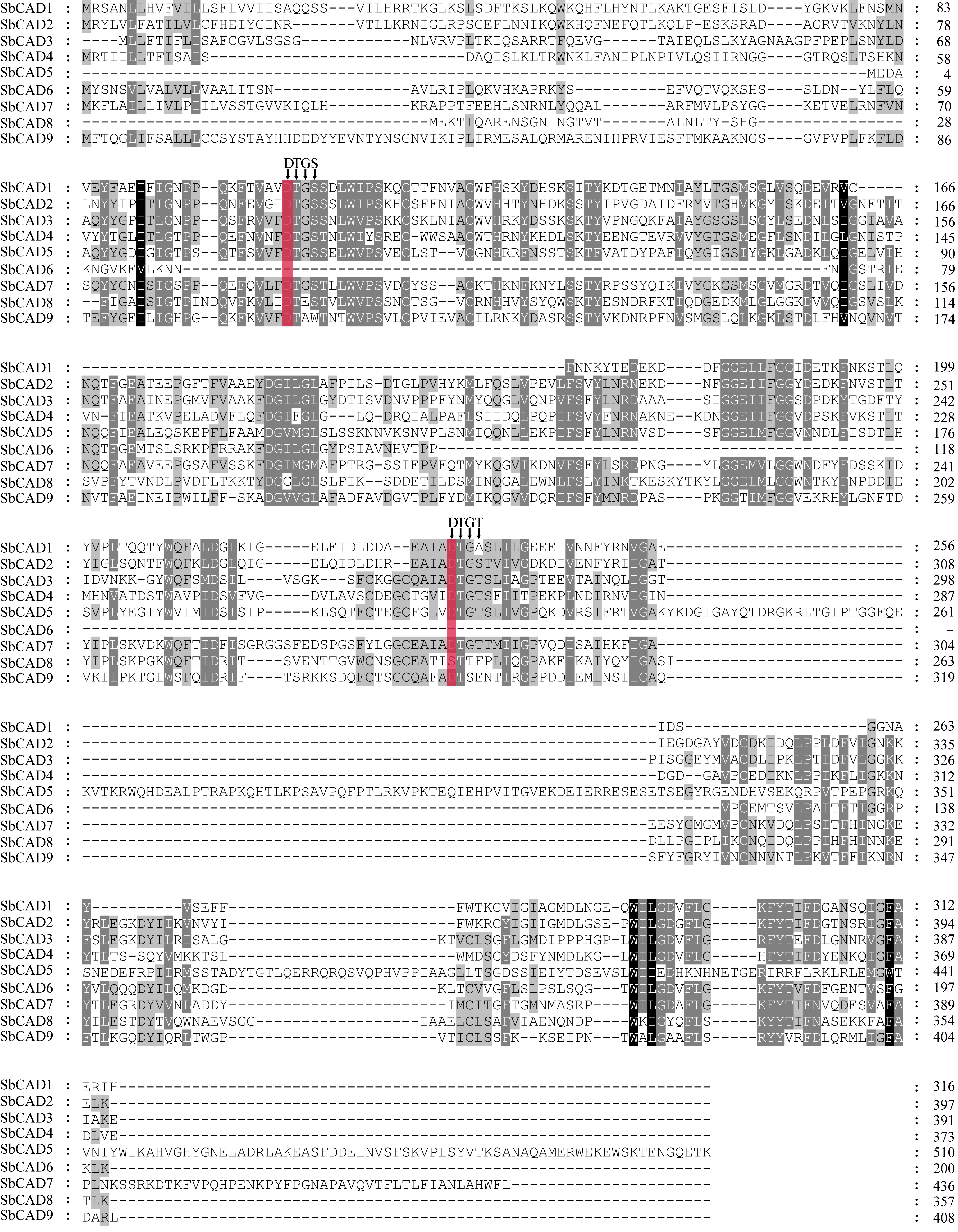

Supplement: Supplementary file 1 [file insects-16-01078-s001.zip › Supplementary Files/Figure S2.docx]

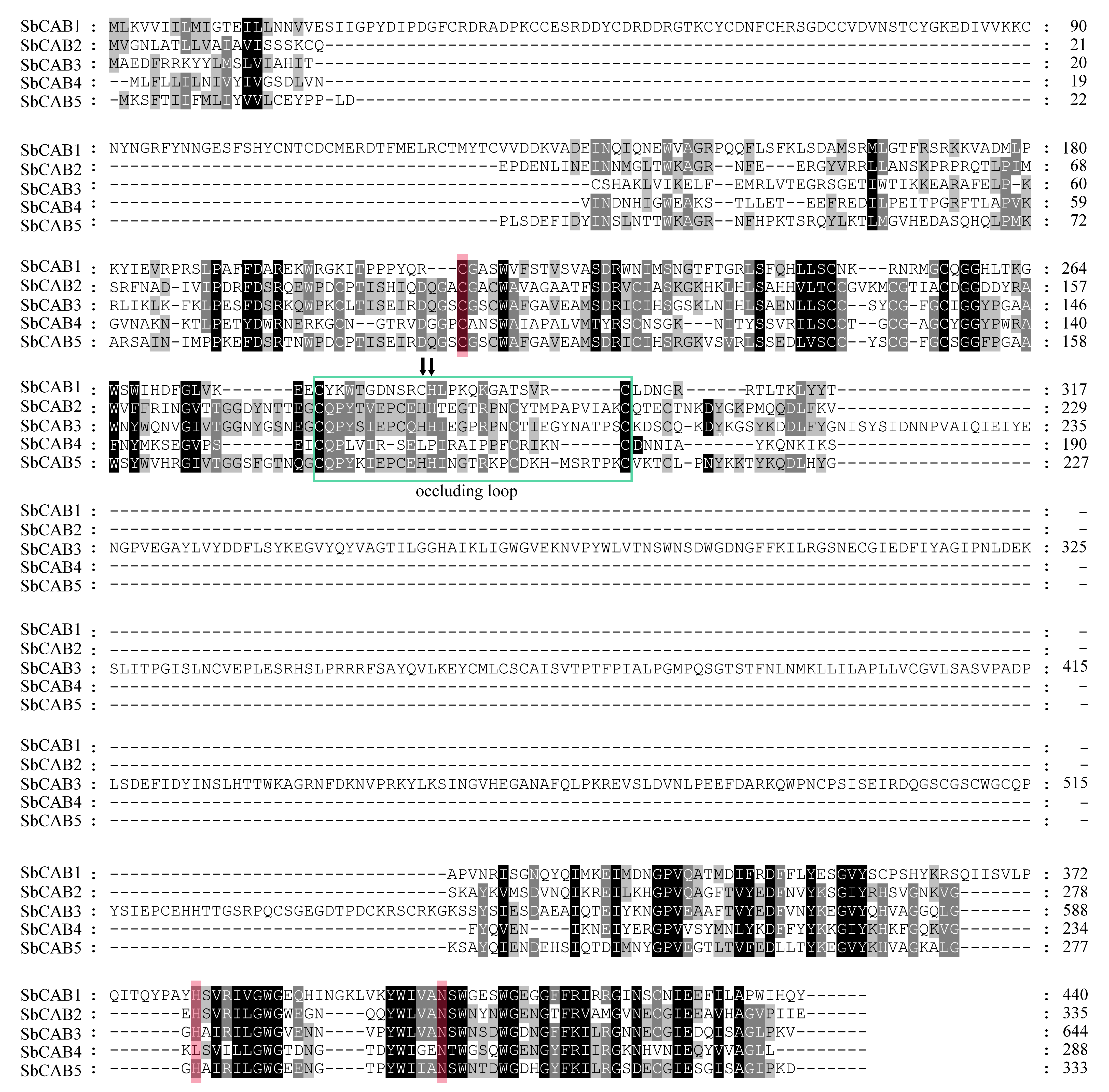

Supplement: Supplementary file 1 [file insects-16-01078-s001.zip › Supplementary Files/Figure S3.docx]

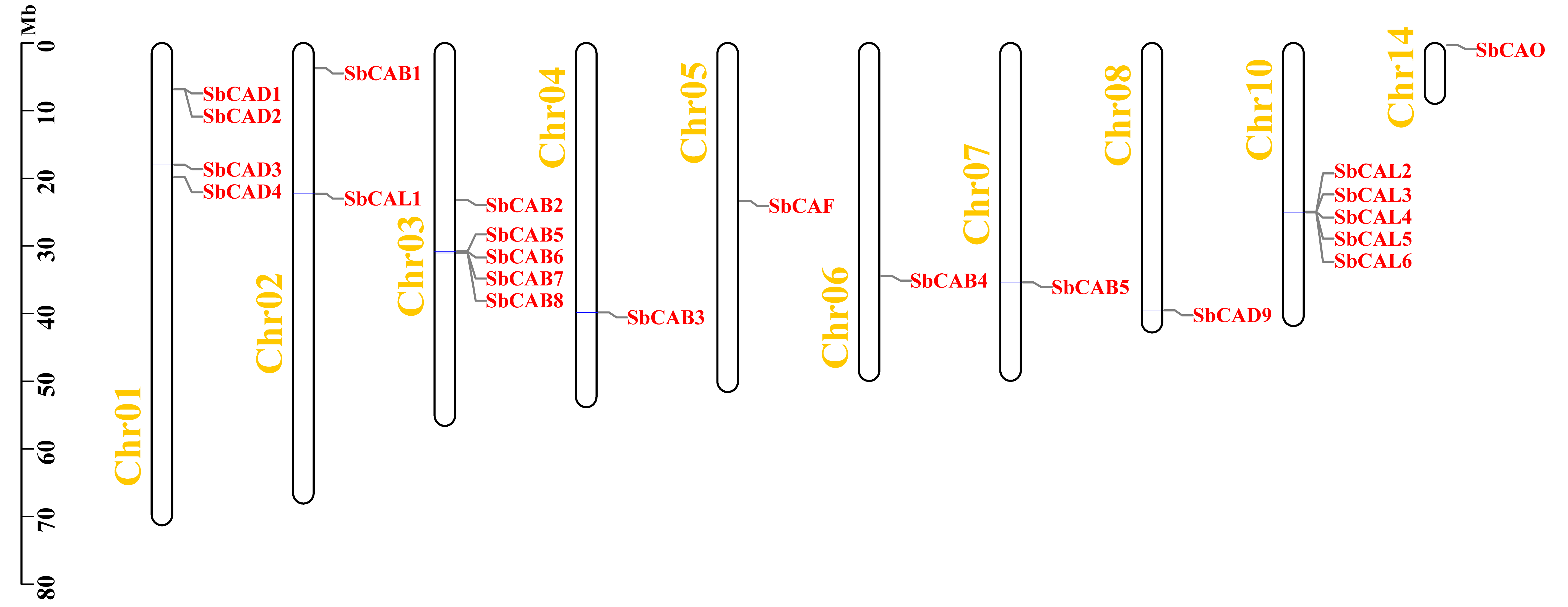

Supplement: Supplementary file 1 [file insects-16-01078-s001.zip › Supplementary Files/Figure S4.docx]
